# FOXA1 and RAB25 as Biomarkers of Breast Cancer Cell Response to CYP1A1-Activated Prodrugs: Insights from CEU-938

**DOI:** 10.3390/ph19030357

**Published:** 2026-02-25

**Authors:** Quentin Bruxelles, Geneviève Hamel-Côté, Marie-Pier Scott-Boyer, Vincent Ouellette, René C.-Gaudreault, Francine Durocher, Caroline Diorio, Arnaud Droit, Sébastien Fortin

**Affiliations:** 1 Faculté de Pharmacie, Université Laval, Québec, QC G1V 0A6, Canada; quentin.bruxelles.1@ulaval.ca (Q.B.); vincent.ouellette.2@ulaval.ca (V.O.); 2 Axe Oncologie, Centre de Recherche du CHU de Québec—Université Laval, Québec, QC G1L 3L5, Canada; genevieve.hamel-cote@crchudequebec.ulaval.ca (G.H.-C.); rene.c-gaudreault@crchudequebec.ulaval.ca (R.C.-G.); caroline.diorio@crchudequebec.ulaval.ca (C.D.); 3 Centre de Recherche sur le Cancer, Université Laval, Québec, QC G1J 0J9, Canada; francine.durocher@crchudequebec.ulaval.ca (F.D.); arnaud.droit@crchudequebec.ulaval.ca (A.D.); 4 Axe Endocrinologie et Néphrologie, Centre de Recherche du CHU de Québec—Université Laval, Québec, QC G1V 4G2, Canada; mariepier.scottboyer@crchudequebec.ulaval.ca; 5 Faculté de Médecine, Département de Médecine Moléculaire, Université Laval, Québec, QC G1V 0A6, Canada; 6 Faculté de Médecine, Département de Médecine Sociale et Préventive, Université Laval, Québec, QC G1V 0A6, Canada

**Keywords:** anticancer agents, antimitotics, antimicrotubule agents, CYP1A1-activated prodrugs, phenyl 4-(2-oxo-3-alkylimidazolidin-1-yl)benzenesulfonates, PAIB-SOs, CEU-938

## Abstract

**Background/Objectives:** CEU-938, an innovative antimicrotubule prodrug bioactivated by cytochrome P450 1A1 (CYP1A1), represents a promising targeted alternative for cancer cells overexpressing this enzyme. To optimize its clinical utility and minimize off-target effects in breast cancer (BC) patients, this study aims to identify predictive biomarkers of CEU-938 efficacy. **Methods:** The antiproliferative activity of CEU-938 was assessed across a panel of 39 human breast cancer and non-tumorigenic cell lines. Differential expression analyses were subsequently performed to distinguish CEU-938-responsive from non-responsive cell lines using a threshold of 1000 nM. Candidate biomarkers identified through this approach were then validated by RT-qPCR and Western blot analyses. **Results:** CEU-938 demonstrated marked and selective antiproliferative activity across molecular subtypes of human breast cancer, with efficacy observed in approximately 40% of triple-negative breast cancer (TNBC), 70% of estrogen receptor-positive (ER^+^), and 80% of human epidermal growth factor receptor 2-positive (HER2^+^) breast cancer cell lines, while sparing non-tumorigenic human breast cells (MCF 10A, MCF-12A, 184B5). Differential expression analysis identified five candidate biomarkers associated with CEU-938 responsiveness, namely, FOXA1 (log2-fold change (LFC) = 3.1), RAB25 (LFC = 3.8), RHOV (LFC = 2.9), PRKCH (LFC = 1.6), and HDAC9 (LFC = −1.7). Among these, FOXA1 and RAB25 robustly validated by RT-qPCR and Western blot analyses, showing strong inverse correlations with CEU-938 sensitivity (Spearman correlation coefficients of −0.82 and −0.61, respectively, at the protein level). The predictive value of FOXA1 and RAB25 was further confirmed by Western blot analyses in two independent breast cell line models, the non-responsive MCF-12A and the responsive MDA-kb2. **Conclusions:** Collectively, these findings identify FOXA1 and RAB25 as robust predictive biomarkers of response to CEU-938. Notably, FOXA1 and RAB25 are strongly implicated in breast cancer biology, and FOXA1 has been directly linked to the aryl hydrocarbon receptor (AHR), the main regulator of CYP1A1. These results position CEU-938 as a strong precision-therapy candidate that combines target selectivity, a favorable toxicity profile, and biomarker-enabled patient stratification, with potential clinical benefit in ER^+^ and HER2^+^ enriched tumors, as well as a subset of TNBC.

## 1. Introduction

In 2022, breast cancer (BC) was the most frequently diagnosed cancer among women in 157 out of 185 countries, with an estimated 2.3 million new cases reported worldwide [1]. While early-stage BC generally responds favorably to standard therapies, a significant subset of cases are intrinsically aggressive, poorly responsive to treatments, or diagnosed at advanced stages. These subtypes are often associated with increased therapeutic resistance, a higher risk of recurrence and metastasis, and an overall unfavorable prognosis [2]. Consequently, many patients require systemic conventional chemotherapy as part of their treatment regimen. However, chemotherapeutic regimens are commonly accompanied by significant adverse effects, including nausea, vomiting, fatigue, and peripheral neuropathy [3]. To overcome these limitations, novel targeted therapies such as in situ enzyme-activated prodrugs have been developed to enhance therapeutic efficacy, improve patients’ quality of life, and minimize adverse effects [4]. This strategy leverages highly cytotoxic compounds that are chemically masked and inactive upon systemic administration. The prodrugs remain inert until they are selectively bioactivated by enzymes present in the tumor microenvironment, triggering the localized release of their active metabolites. By restricting activation to the tumor site, this approach can increase cytotoxic impact in cancer cells while sparing healthy tissues [5]. In this context, enzyme-activated prodrugs offer a promising therapeutic option for targeting advanced stages and drug-resistant BC, with the potential to improve efficacy while limiting systemic toxicity.

**Figure 1:**
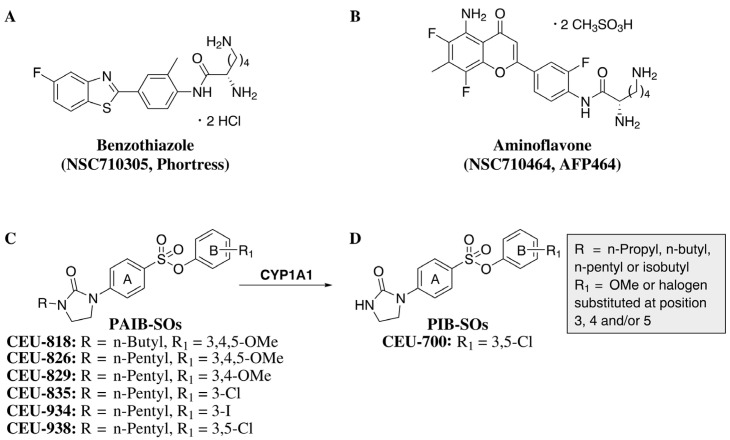
Molecular structures of CYP1A1-dependent prodrugs. (**A**) Benzothiazole (2S)-2,6-diamino-*N*-[4-(5-fluoro-2-benzothiazolyl)-2-methylphenyl] hexanamide dihydrochloride (NSC710305, Phortress), (**B**) aminoflavone (2S)-2,6-diamino-*N*-[4-(5-amino-6,8-difluoro-7-methyl-4-oxochroman-2-yl)-2-fluorophenyl] hexanamide dimethanesulfonate (NSC710464, AFP464) and (**C**) phenyl 4-(2-oxo-3-alkylimidazolidin-1-yl)benzenesulfonates (PAIB-SOs). Bioactivation of PAIB-SOs into (**D**) antimitotic phenyl 4-(2-oxoimidazolidin-1-yl)-benzenesulfonate (PIB-SO) by CYP1A1 ((**C**) to (**D**)).

Cytochrome P450 1A1 (CYP1A1) and its associated signaling pathway play a pivotal role in the detoxification of xenobiotics, including polycyclic aromatic hydrocarbons and a wide range of environmental pollutants. Importantly, CYP1A1 is overexpressed in several cancer subtypes, such as breast, lung, gastrointestinal, and ovarian cancers, underscoring its relevance as a therapeutic target for enzyme-activated prodrug strategies [6,7,8,9]. In recent years, CYP1A1-activated prodrugs benzothiazole (2S)-2,6-diamino-*N*-[4-(5-fluoro-2-benzothiazolyl)-2-methylphenyl] hexanamide dihydrochloride (NSC710305, Phortress, Figure 1A) and aminoflavone (2S)-2,6-diamino-*N*-[4-(5-amino-6,8-difluoro-7-methyl-4-oxochroman-2-yl)-2-fluorophenyl] hexanamide dimethanesulfonate (NSC710464, AFP464, Figure 1B), both precursors of DNA alkylating agents, have been developed and evaluated in clinical studies [10,11]. However, the clinical development of these compounds was ultimately discontinued due to unacceptable adverse effects, notably pulmonary toxicity [12,13]. To overcome this limitation, we developed a novel class of CYP1A1-activated prodrugs named phenyl 4-(2-oxo-3-alkylimidazolidin-1-yl)benzenesulfonates (PAIB-SOs, Figure 1C) engineered to specifically release potent antimitotic agents through *N*-dealkylation. The resulting microtubule-targeting metabolites, referred to as phenyl 4-(2-oxoimidazolidin-1-yl)benzenesulfonates (PIB-SO, Figure 1D), display improved tolerability relative to classical alkylating agents, show no evidence of pulmonary toxicity, and appear to remain unaffected by major chemoresistance mechanisms studied so far [14]. Moreover, these compounds bind strongly to the colchicine-binding site on tubulin, disrupt microtubule dynamics, and induce G2/M cell-cycle arrest [15]. Through extensive screening and biofunctional assays, 3,5-dichlorophenyl 4-(2-oxo-3-pentylimidazolidin-1-yl)benzenesulfonate (CEU-938) was selected as the leading prodrug. CEU-938 undergoes CYP1A1-mediated *N*-dealkylation to release 3,5-dichlorophenyl-4-(2-oxoimidazolidin-1-yl)benzenesulfonate (CEU-700), its active antimitotic metabolite [16]. Notably, CEU-938 showed potent antitumor activity with no detectable adverse effects at the tested doses [17]. Collectively, these findings position CEU-938 as the first CYP1A1-activated prodrug that delivers antimitotic activity and represents a promising strategy to reduce treatment-related toxicity.

Another complementary strategy to minimize adverse effects, enhance therapeutic efficacy, and mitigate chemoresistance is the prediction of treatment response. A widely used approach involves identifying patients who express biomarkers predictive of drug sensitivity [18]. Notably, several biomarkers, including forkhead box protein A1 (FOXA1), a transcription factor, and Ras-related protein 25 (RAB25), a small GTPase, are already used in clinical and translational contexts for tumor characterization and patient stratification [19,20]. We therefore hypothesized that bioinformatics analysis based on transcriptomic features would enable the identification of molecular biomarkers associated with and predictive of CEU-938 therapeutic response. To test this hypothesis, we evaluated the antiproliferative activity of CEU-938 across a panel of human breast cancer and non-tumorigenic breast cell lines. Based on their differential sensitivity profiles, we performed bioinformatics analyses to identify candidate response-associated biomarkers, which were subsequently validated by RT-qPCR and Western blotting. Finally, their predictive value was independently confirmed in two additional human breast cell lines by Western blots.

## 2. Results

### 2.1. Antiproliferative Activity (IC_50_) of CEU-938 in a Panel of Human Breast Cell Lines

**Figure 2:**
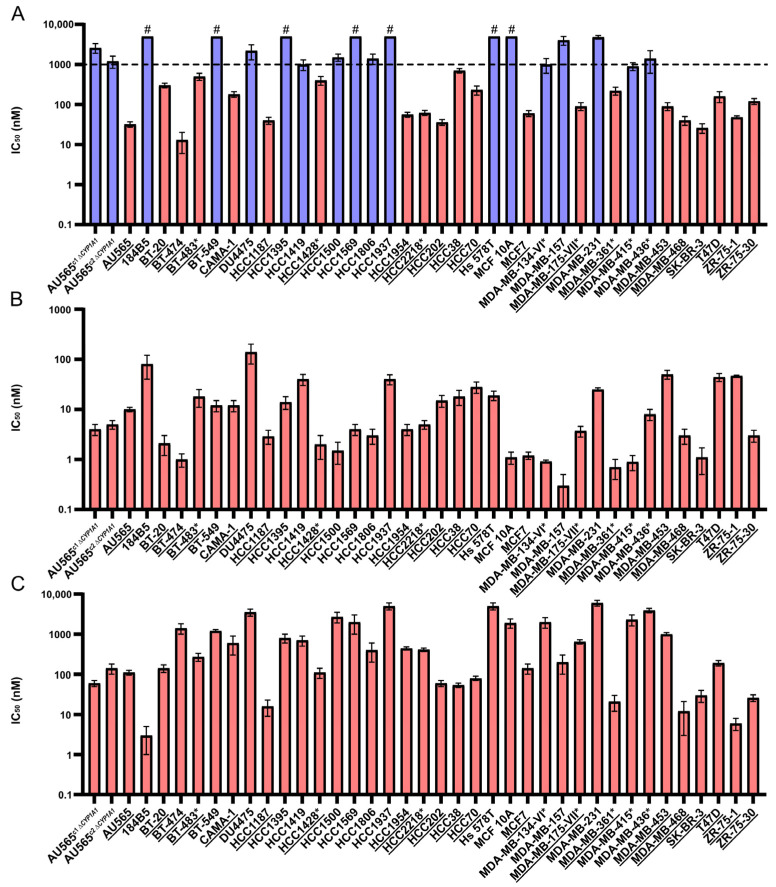
CEU-938 activity across a panel of 37 human breast cancers (BC) and non-tumorigenic cell lines (21 responsive cell lines). (**A**) Antiproliferative activity (IC_50_) of CEU-938 was determined in 37 cell lines using either the sulforhodamine B (SRB) assay for adherent cells or the lactate dehydrogenase (LDH) assay for non-adherent cells, with escalating concentrations up to the solubility limit. Responsive (pink, underlying) and non-responsive (blue) cell lines are indicated. (**B**) IC_50_ values for CEU-700. (**C**) IC_50_ values for Phortress. CEU-700, the active antimicrotubule metabolite of CEU-938, and Phortress, a CYP1A1-activated DNA alkylating prodrug previously evaluated in clinical trials, were used as positive and reference controls, respectively. DMSO (0.5%) was used as a negative control. IC_50_ values were determined at 48 h, except for cell lines marked with an asterisk (*), which were assessed at 96 h due to slower proliferation. Cell lines marked with a hash (#) exhibited IC_50_ values above the solubility limit of the tested compounds. Bars represent mean values ± SEM from at least three independent experiments performed in triplicate. The underlying cell-line model was defined based on CEU-938-responsive cell lines. The dashed line indicates the sensitivity cutoff at 1000 nM used to discriminate responsive from non-responsive cell lines.

Our earlier work established that CEU-938 is a potent antimicrotubule prodrug bioactivated by CYP1A1, resulting in selective antiproliferative activity in CYP1A1-positive BC cells while exerting minimal impact on CYP1A1-deficient BC cell lines [17]. To further evaluate its anticancer activity and therapeutic relevance across diverse BC subtypes, we assessed CEU-938 in a panel of 39 human BC and non-tumorigenic breast cell lines (Supplementary Table S1). For comparison, CEU-700 (the active antimicrotubule metabolite of CEU-938) and Phortress (a CYP1A1-activated alkylating prodrug evaluated clinically), as well as paclitaxel and vinblastine, were included as positive controls. To experimentally confirm the role of CYP1A1 in prodrug activation and to prespecify a sensitivity threshold prior to bioinformatics analyses, AU565 CYP1A1-knockout (AU565*^ΔCYP1A1^*) clones were generated by CRISPR/Cas9 and evaluated for drug responsiveness. At 48 and 96 h, CEU-938 exhibits IC_50_ values of 2000 and 2600 nM in AU565^c1 *ΔCYP1A1*^ cells and 4000 and 1200 nM in AU565^c2 *ΔCYP1A1*^, compared to 32 and 32 nM in AU565 wild-type (Table 1 and Table 2). These results correspond to a selectivity ratio ranging from 38 to 125-fold, indicating that concentrations above this range may trigger CYP1A1-independent mechanisms. Based on these results, we established a threshold to define cellular sensitivity to CEU-938 using the response observed in AU565*^ΔCYP1A1^* at 96 h, which provided improved discrimination from potential alternative pathways. On this basis, a cut-off value of 1000 nM was established to define cellular sensitivity, as this concentration was both biologically justified and consistent with pharmacologically achievable in vivo exposure levels. To minimize off-target effects and to emphasize CYP1A1 dependence, we preferentially used the 48 h IC_50_ values when available. Antiproliferative activity varied across compounds, with IC_50_ values ranging from 13 to >5000 nM for CEU-938, 0.3 to 140 nM for CEU-700, 3 to 6000 nM for Phortress, 0.13 to 40 nM for paclitaxel and 0.14 to 60 nM for vinblastine, respectively (Figure 2A–C and Table 1). Using the 1000 nM cutoff, 21 cell lines were classified as responsive and 16 as non-responsive, the latter group including the three non-tumorigenic cell lines. Overall, this corresponds to 62% of breast cancer cell lines being classified as responsive. Notably, CEU-938 responsiveness was observed in 40% of triple-negative breast cancer (TNBC), 70% of estrogen receptor-positive (ER^+^), and 80% of HER2-positive (HER2^+^) cell lines (Supplementary Table S1), based on the BC classification reported by Smith et al. [21]. As expected, CEU-700, the active antimicrotubule metabolite of CEU-938, displayed similar IC_50_ values in AU565*^ΔCYP1A1^* and wild-type cells, confirming its CYP1A1-independent activity. Moreover, CEU-700, paclitaxel and vinblastine were active across all cell lines tested, further confirming their broad antiproliferative profile. In contrast, the CYP1A1-activated alkylating prodrug Phortress exhibited only minimal CYP1A1 specificity, as evidenced by comparable IC_50_ values in both AU565*^ΔCYP1A1^* (60–140 nM) and wild-type AU565 cells (91 nM) at 96 h. At 48 h, however, Phortress exhibited IC_50_ values ranging from 500 to 1000 nM in AU565*^ΔCYP1A1^* clones versus 110 nM in AU565 wild-type cells, corresponding to a 4.5–9.0-fold selectivity window. Based on these results, a sensitivity threshold of 1000 nM was established for Phortress sensitivity (Supplementary Figure S1). Using this threshold, 24 cell lines were classified as Phortress-responsive, including 50% of TNBC, 70% of ER^+^, and 80% HER2^+^ breast cancer cell lines (Supplementary Table S1). Notably, nearly 80% of cell lines (30/39) displayed concordant response profiles to both CEU-938 and Phortress. Testing at 96 h gave a comparable responsive/non-responsive classification for most cell lines (Table 2).

### 2.2. FOXA1, RAB25, RHOV, PRKCH, and HDAC9 as Candidate Biomarkers for CEU-938-Sensitive BC Cells

**Figure 3:**
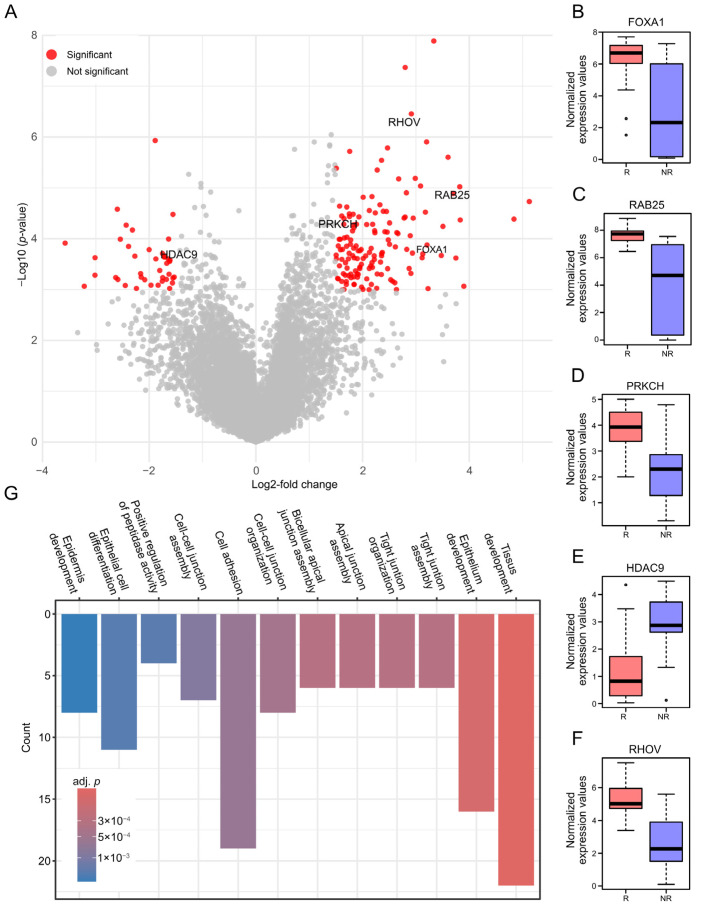
Bioinformatics analysis of 37 human BC cell lines, stratified by CEU-938 IC_50_ values, identifies FOXA1, RAB25, RHOV, PRKCH, and HDAC9 as candidate response-associated biomarkers. (**A**) Volcano plot showing 183 differentially expressed genes between CEU-938-responsive and non-responsive cell lines, identified through PCA of CEU-938 IC_50_ values (log2-fold change (LFC) ≥ 1.5). Normalized gene expression profile (log-transformed transcripts per million values) comparing responsive (R, pink) and non-responsive (NR, blue) cell lines for (**B**) FOXA1, (**C**) RAB25, (**D**) PRKCH, (**E**) HDAC9 and (**F**) RHOV. (**G**) Functional enrichment analysis of the 183 differentially expressed genes (stringent LFC cutoff ≥ 2).

To investigate molecular differences between CEU-938-responsive and non-responsive BC cell lines, we performed differential gene expression analysis using RNA-sequencing data from the cell lines evaluated above (Supplementary Table S1), leveraging the dataset reported by Ghandi et al. [22]. Principal component analysis (PCA, Supplementary Figure S2) revealed separation between the two groups along the first two principal components, consistent with distinct global transcriptional profile differences in their overall gene expression patterns. Overall, 183 genes were differentially expressed and visualized using a volcano plot, applying a log2-fold change (LFC) threshold of 1.5 (Figure 3A). Subsequent filtering identified five candidate biomarkers. Candidate selection was guided by multiple criteria, including the magnitude of the LFC, the adjusted *p*-value (adj. *p*), biological relevance based on the literature, and reported links to the aryl hydrocarbon receptor (AHR)-CYP1A1 signaling axis. Notably, FOXA1, which has been reported to functionally interact with AHR, emerged among the top candidates and was significantly upregulated (LFC = 3.1, adj. *p* = 0.026, Figure 3B) [23]. RAB25 (Ras-related protein 25), involved in cancer progression and frequently associated with FOXA1 in published studies, also exhibited strong upregulation (LFC = 3.8, adj. *p* = 0.006, Figure 3C) [24,25]. PRKCH (Protein kinase C eta), a member of the PKC family with reported involvement in AHR signaling, showed moderate upregulation (LFC = 1.6, adj. *p* = 0.01, Figure 3D) [26]. In contrast, HDAC (histone deacetylase 9) was significantly downregulated (LFC = −1.7, adj. *p* = 0.03, Figure 3E), consistent with evidence suggesting that histone deacetylases can modulate the AHR-CYP1A1 signaling pathway [27]. Despite no direct link having been reported between Ras homolog family member V (RHOV) and the AHR-CYP1A1 signaling pathway, this gene was prioritized based on its strong upregulation (LFC = 2.9, adj. *p* = 0.002, Figure 3F), prior evidence implicating RHOV in TNBC, and its potential role in cytoskeletal regulation. This is particularly relevant, considering the mechanism of action of CEU-700, the antimicrotubule metabolite of CEU-938 generated through CYP1A1-mediated bioactivation, which is expected to intersect with pathways controlling cytoskeletal dynamics [28,29]. To further characterize the biological processes associated with the differentially expressed genes and CEU-938 responsiveness, we subsequently performed gene set enrichment analysis. To enhance biological relevance, a more stringent LFC cutoff of 2 was applied to downstream analyses. Gene set enrichment of pathways related to tissue and epithelium development, cell adhesion, membrane dynamics, and cell junction organization features consistent with the cytoskeletal and adhesion processes that can be perturbed by microtubule-targeting agents, in line with CEU-938 activity via its antimicrotubule metabolite CEU-700 (Figure 3G). Altogether, these analyses provide insight into the molecular features associated with CEU-938 responsiveness and identify FOXA1, RAB25, RHOV, PRKCH and HDAC9 as candidate biomarkers predictive of cellular sensitivity to CEU-938.

### 2.3. FOXA1 and RAB25 as Validated Predictive Biomarkers of CEU-938 Antiproliferative Activity

**Figure 4:**
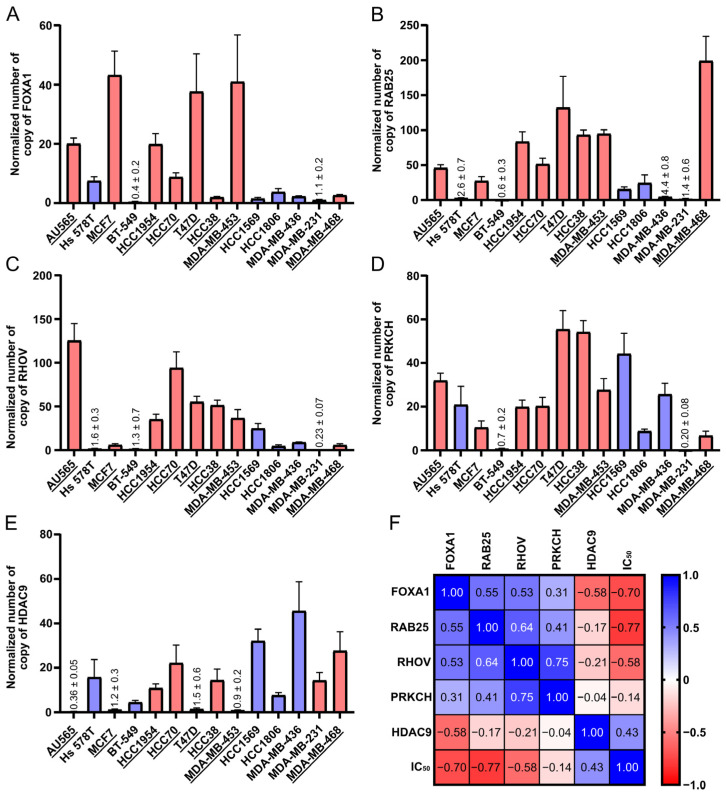
Correlation between FOXA1, RAB25, RHOV, and HDAC9 mRNA expression and CEU-938 IC_50_ values. RT-qPCR analysis of (**A**) FOXA1, (**B**) RAB25, (**C**) RHOV, (**D**) PRKCH, and (**E**) HDAC9 in a subset of 14 breast cancer cell lines classified as responsive (R, pink, underlying) or non-responsive (NR, blue). The panel comprised nine triple-negative breast cancer (TNBC: BT-549 (NR), HCC1806 (NR), HCC38 (R), HCC70 (R), HS578T (NR), MDA-MB-231 (NR), MDA-MB-436 (NR), MDA-MB-453 (R), MDA-MB-468 (R), three HER2-positive HER2^+^: AU565 (R), HCC1569 (NR), HCC1954 (R), and two estrogen receptor-positive (ER^+^): MCF7 (R), T47D (R). (**F**) Spearman correlation analysis between mRNA expression levels of FOXA1, RAB25, RHOV, PRKCH, and HDAC9 and CEU-938 sensitivity (IC_50_). Bars represent the mean ± SEM of four independent experiments performed in triplicate.

Following the identification of FOXA1, RAB25, RHOV, PRKCH, and HDAC9 as candidate genes potentially associated with the antiproliferative activity of CEU-938, we performed experimental validation across a panel of 14 BC cell lines (Figure 4A–F). Cell lines were selected to span a wide range of CEU-938 sensitivities (IC_50_ ranging from 32 nM to >5000 nM), including 8 responsive and 6 non-responsive models, and to represent the major receptor-defined BC subtypes (9 TNBC, 3 HER2^+^, and 2 ER^+^). RT-qPCR analysis showed that FOXA1 expression was significantly higher in CEU-938-sensitive cell lines (Figure 4A) compared to less responsive models and was strongly inversely correlated with IC_50_ values (r = −0.70, *p* = 0.0065). Similarly, RAB25 and RHOV were also upregulated in the most responsive cell lines (Figure 4B,C) and exhibited significant negative correlations with IC_50_ values (r = −0.77, *p* = 0.002 and r = −0.58, *p* = 0.033, respectively). PRKCH expression remained relatively constant across the panel and showed no apparent association with CEU-938 activity (Figure 4D). In contrast, HDAC9 expression tended to be higher in the less responsive cell lines (Figure 4E), although its positive correlation with IC_50_ (r = 0.43) did not reach statistical significance (*p* = 0.122). Collectively, these results identify FOXA1, RAB25, and RHOV as strong predictors of CEU-938 responsiveness, with HDAC9 showing moderate predictive potential, thereby supporting further mechanistic investigation.

**Figure 5:**
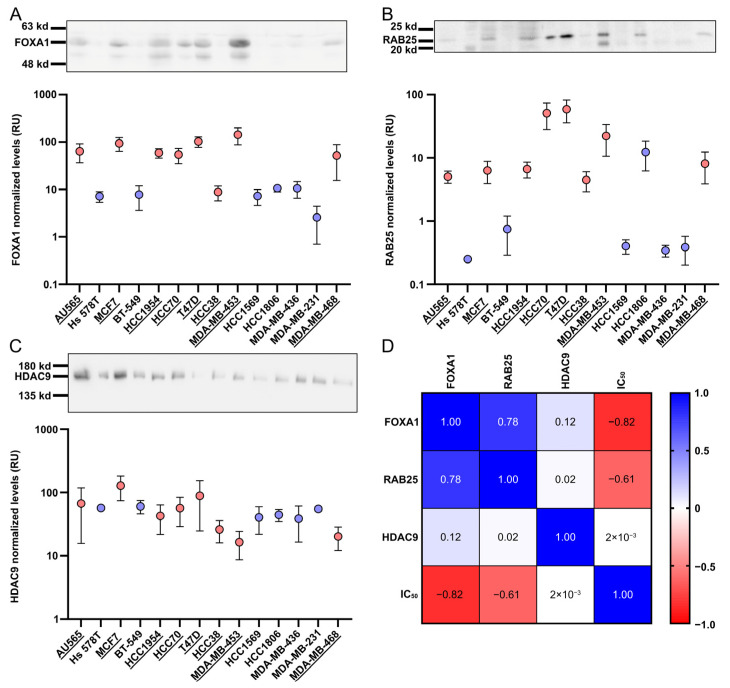
FOXA1 and RAB25 protein levels correlate with CEU-938 IC_50_ values. Western blot analysis of (**A**) FOXA1, (**B**) RAB25, and (**C**) HDAC9 expression in a subset of 14 breast cancer (BC) cell lines classified as CEU-938-responsive (pink, underlying) and non-responsive (blue). (**D**) Spearman correlation analysis between FOXA1, RAB25, and HDAC9 protein expression levels and CEU-938 IC_50_ values. Bars represent mean ± SEM from three independent experiments performed in triplicate.

To further substantiate these findings, FOXA1, RAB25, RHOV, and HDAC9 were examined at the protein level by Western blotting (Figure 5, Supplementary Figures S3 and S4). FOXA1 protein expression was higher in CEU-938-responsive cell lines (Figure 5A) and showed a strong inverse correlation with IC_50_ values (r = −0.82, *p* = 0.001; Figure 5D). This observation was independently confirmed using a second antibody (Supplementary Figure S5). Similarly, RAB25 protein levels were elevated in the more responsive cell lines (Figure 5B) and correlated negatively with IC_50_ values (r = −0.61, *p* = 0.024). In contrast, HDAC9 protein expression was not enriched in non-responsive cell lines and exhibited no correlation with CEU-938 sensitivity (Figure 5C), suggesting limited predictive value at the protein level. Moreover, despite testing multiple antibodies against RHOV (Supplementary Figure S4), we did not detect a band corresponding to its expected molecular weight (~26 kDa). Although, a signal was consistently detected at approximately 55 kDa and broadly mirrored the RHOV pattern, this apparent molecular weight is inconsistent with the predicted size of RHOV, suggesting that the detected band most likely reflects antibody cross-reactivity rather than specific protein detection in Western blots. Taken together, these data validate FOXA1 and RAB25 as robust predictive biomarkers of CEU-938 activity at both the transcript and protein levels. In contrast, HDAC9 appears to have a predictive value primarily at the mRNA level. The biomarker potential of RHOV remains inconclusive due to the lack of reliable protein detection. Nevertheless, it may still warrant consideration as an mRNA-based marker pending further investigation.

### 2.4. FOXA1 and RAB25 Confirmed as Predictive Biomarkers of CEU-938 Efficacy Across Independent Breast Cell Lines

**Figure 6:**
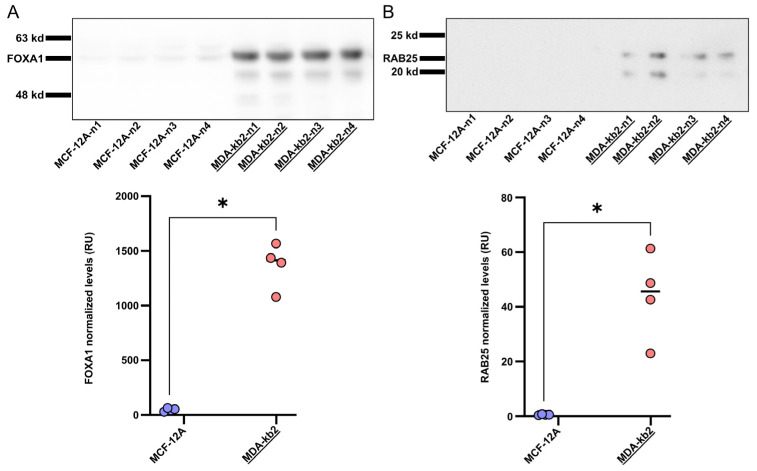
Independent Validation of FOXA1 and RAB25 as predictive biomarkers in human breast cell lines. Western blot and quantification of protein expression in non-responsive MCF-12A (blue) and responsive MDA-kb2 (pink) of (**A**) FOXA1 and (**B**) RAB25. Statistical significance was assessed using Student’s *t*-test (* *p* < 0.05).

To further evaluate the predictive value of FOXA1 and RAB25 beyond transcriptomic analysis, we assessed CEU-938 antiproliferative activity together with FOXA1 and RAB25 protein expression in two additional breast-derived cell lines not included in the differential expression analysis: MCF-12A and MDA-kb2. IC_50_ determination revealed a marked difference in sensitivity, with MDA-kb2 cells displaying high sensitivity (IC_50_ = 30 nM), whereas MCF-12A cells were strongly resistant (IC_50_ > 5000 nM). Consistent with these divergent response profiles, Western blotting analysis showed significantly higher FOXA1 protein levels (Figure 6A, Supplementary Figure S6A) and RAB25 protein levels (Figure 6B, Supplementary Figure S6B) in MDA-kb2 compared with MCF-12A. Collectively, these results provide independent validation of FOXA1 and RAB25 as robust predictive biomarkers of CEU-938 responsiveness, including at the protein level.

### 2.5. Extension of the Use of FOXA1 and RAB25 as Biomarkers to Others PAIB-SO Molecules

The PAIB-SOs family includes several potent prodrugs that could serve as alternatives to overcome potential challenges encountered during the preclinical or clinical development of CEU-938 [15,17,30]. To explore this possibility, we compared CEU-938 with additional promising PAIB-SO derivatives, including CEU-818, CEU-826, CEU-829, CEU-835, and CEU-934. Their antiproliferative activity was assessed across the same panel of BC cell lines used for CEU-938. IC_50_ determination showed that 72 to 85% of the cell lines displayed response profiles similar to CEU-938 (Table 3). Furthermore, within the subset of cells selected for RT-qPCR and Western blot analyses, IC_50_ values exhibited 79 to 86% concordance with CEU-938. These results suggest that FOXA1 and RAB25 may serve as predictive biomarkers not only for CEU-938 but also more broadly across the PAIB-SO prodrug family.

## 3. Discussion

This study outlines an approach most likely to benefit from CEU-938 by refining stratification criteria, thereby maximizing therapeutic efficacy while minimizing the risk of deleterious effects. Accordingly, prior to initiating bioinformatics analyses, we first defined an IC_50_ sensitivity threshold using AU565*^ΔCYP1A1^* cells. Our results show that CEU-938 remains CYP1A1-dependent at concentrations of up to 1000 nM, underscoring the pivotal role of CYP1A1 in its mechanism of action. We acknowledge, however, that cell lines with IC_50_ values close to this cutoff may be susceptible to minor experimental variability, which could affect their sensitivity response classification and, consequently, influence the set of differentially expressed genes identified. This potential limitation should, therefore, be considered when interpreting borderline cases. Using this threshold, 22 of the 39 BC cell lines tested were classified as CEU-938-responsive, including seven TNBC cell lines. Conversely, 17 breast cell lines were classified as non-responsive, including the three non- tumorigenic breast epithelial lines 184B5, MCF 10A, and MCF-12A. The lower representation of TNBC cell lines within the responsive group likely reflects a combination of biological and sampling-related factors. Biologically, several TNBC models exhibit lower CYP1A1 and FOXA1 expression levels, which may limit their sensitivity to CEU-938 given the CYP1A1 dependent mechanism of action identified in this study. In addition, TNBC represents the largest subgroup in our panel, thereby increasing the probability that a greater absolute number of TNBC cell lines will be represented among the non-responsive models. This distribution should, therefore, be interpreted cautiously and not solely attributed to intrinsic subtype resistance. By contrast, standard antimicrotubule agents such as paclitaxel and vinblastine exhibit broadly indiscriminate cytotoxicity across the panel of human breast cell lines, including non-cancerous cells. Together, these results suggest that CEU-938 may represent a more selective therapeutic option for hard-to-treat cancers while sparing healthy tissues, potentially reducing systemic toxicity. Moreover, the antiproliferative activity profiles of other PAIB-SOs parallel those of CEU-938, supporting the notion that these compounds share overlapping mechanisms of action. Consequently, these analogs may also prove effective in patients whose molecular profiles predict responsiveness to CEU-938, thereby broadening therapeutic opportunities across this family of compounds.

Based on CEU-938 antiproliferative activity profile, we conducted a differential gene-expression analysis that identified 183 genes distinguishing CEU-938-responsive (CYP1A1-dependent) from non-responsive cell lines. Of these, 143 genes were enriched in responsive cell lines and 40 in non-responsive cell lines. Subsequent gene set enrichment analysis highlighted several signaling pathways related to plasma membrane organization, cell adhesion, and cell migration, which is consistent with the microtubule-disrupting activity of the active metabolite generated from CEU-938 [31]. Collectively, these findings suggest that CEU-938 may preferentially target cells characterized by heightened intracellular trafficking and dynamic cytoskeletal remodeling, features typical of BC cells, while sparing non-tumorigenic breast epithelial cell lines such as 184B5, MCF 10 A and MCF-12A.

To guide biomarker selection for CEU-938 efficacy, we prioritized genes among the 183 differentially expressed candidates that are functionally linked to the CYP1A1 signaling pathway. This strategy was motivated by previous evidence showing that PAIB-SOs, including CEU-938, require CYP1A1-mediated metabolism to generate antimicrotubule-active PIB-SOs such as CEU-700 [16]. We also included the AHR pathway into our selection criteria, given its established relevance to CYP1A1-targeted prodrugs such as Phortress, which binds AHR and strongly induces CYP1A1 expression.

This interaction establishes a self-reinforcing feedback loop that sustains its CYP1A1-dependent metabolism [32]. Similarly, aminoflavones constitute another family of CYP1A1-dependent prodrugs that rely on CYP1A1 for metabolic activation and can further potentiate their effects through AHR binding [10]. Notably, nearly 80% of the cell lines exhibited comparable response profiles to both CEU-938 and Phortress. This overlap supports the possibility that CEU-938 may engage similar regulatory mechanisms. Guided by this rationale, we selected five promising candidate predictive biomarkers: FOXA1, RAB25, RHOV, PRKCH, and HDAC9. Collectively, these results underscore the importance of integrating both CYP1A1 and AHR signaling pathways when identifying predictive biomarkers for CYP1A1-dependent prodrugs.

FOXA1 was prioritized based on its strong enrichment in CEU-938-responsive cell lines. Notably, FOXA1 has been reported to functionally interact with AHR and to contribute to the regulation of cyclin G2, a pathway implicated in antiproliferative responses [23]. Together, these observations support FOXA1 as a compelling biomarker associated with CEU-938 antiproliferative activity. Although FOXA1 is classically linked to ER^+^ disease and is upregulated in nearly 84% of ER^+^ BCs [33], our results indicate that its predictive value for CEU-938 response is not restricted to ER status. Indeed, HER2^+^ models were also enriched among responsive cell lines, suggesting that FOXA1-related sensitivity extends beyond the ER^+^ subtype. These findings support the potential role of FOXA1 as an independent predictive biomarker. Nevertheless, an ER-related contribution cannot be fully excluded, given the known functional interplay between FOXA1 and ER-dependent signaling pathways. Collectively, these observations suggest that CEU-938 may represent a promising therapeutic option across a broad spectrum of patients.

RAB25 was also strongly enriched in CEU-938-responsive cell lines. This protein has been reported to promote cancer cell invasiveness through the ß1 integrin/EGFR/VEGF-A/Snail signaling axis [34]. Consistently, our bioinformatics analyses revealed significant enrichment of pathways related to integrins [35], EGFR [36], VEGF [37] and Snail [38], including processes involved in cell–cell junction assembly, junction organization, and cell adhesion. Moreover, RAB25 has been reported to be upregulated in radioresistant lung cancer cell lines, and its functional interplay with EGFR has been associated with poor prognosis in patients with lung adenocarcinoma and nasopharyngeal carcinoma [39]. Taken together, these findings support RAB25 as a promising predictive biomarker and raise the possibility that CEU-938 may offer therapeutic benefits to selected subsets of radioresistant lung cancers.

RHOV emerged as one of the most significantly enriched genes in CEU-938-responsive cell lines. Clinically, elevated RHOV expression has been associated with poor clinical outcomes, and functional studies have demonstrated that its knockdown in TNBC cells markedly reduces invasion, migration, and metastatic potential in both in vitro and in vivo models [28]. Together, these findings suggest that RHOV expression may help identify patients with aggressive, treatment-refractory tumors who may particularly benefit from CEU-938-based therapy strategies.

In contrast, HDAC9 was found predominantly enriched in CEU-938-non-responsive cell lines. A functional interplay between histone HDACs and AHR signaling has been documented, notably in the context of AHR activation by gut microbiota-derived ligands [27]. In addition, HDAC9 has been implicated in BC tumorigenesis [40], further supporting its role in oncogenic processes. Altogether, these findings suggest that HDAC9 may serve as a negative predictive biomarker, helping to identify tumors unlikely to benefit from CEU-938 therapy.

Differential expression analysis also identified a significant enrichment of PRKCH in CEU-938-responsive cell lines. PRKCH has been implicated in oncogenic processes across multiple cancer types, where it contributes to tumor progression [41]. Notably, the PKC family, to which PRKCH belongs, has been functionally associated with the AHR signaling pathway, particularly through the induction of CYP1A1 [26,42]. These observations suggest a potential mechanistic connection between PRKCH expression and AHR pathway modulation in the context of CEU-938 responsiveness. However, RT-qPCR analysis did not confirm its enrichment in CEU-938-responsive versus non-responsive cells. Consequently, PRKCH was not retained as a candidate biomarker and will not be further discussed.

RT-qPCR and Western blot analyses consistently confirmed that FOXA1 and RAB25 are enriched in CEU-938-responsive compared with non-responsive cell lines. Their expression levels correlated strongly with CEU-938 antiproliferative activity, and their predictive value was further supported by consistent results obtained in additional cell lines not included in the initial differential expression analysis. Moreover, FOXA1 expression in responsive cell lines was independently validated by Western blot using a second antibody from a different commercial source, confirming the reproducibility of the findings and further reinforcing the robustness of FOXA1 as a predictive biomarker. Although FOXA1 and RAB25 are not directly implicated in CYP1A1 regulation, we hypothesize that their enrichment nonetheless reflects a potential connection to the mechanism of action of CEU-938. For FOXA1, this link may occur indirectly through the AHR pathway, the main regulator of CYP1A1. In contrast, RAB25 is more likely to act as a downstream effector that modulates the cellular response independently of CYP1A1 regulation.

RHOV exhibited promising transcript-level expression by RT-qPCR. However, currently available antibodies failed to produce conclusive signals in Western blots, underscoring the need for further optimization of protein detection strategies. Despite this limitation, RHOV remains a compelling candidate that warrants continued evaluation as a potential predictive biomarker of CEU-938 responsiveness. By contrast, HDAC9 currently appears reliable only at the transcript level, supporting its use as an mRNA-based indicator of CEU-938 non-responsiveness, particularly when integrated with other biomarkers. Finally, other PAIB-SO derivatives and Phortress exhibited up to 86% concordance in response profiles compared to CEU-938. Together, these findings highlight the broader applicability of FOXA1 and RAB25 as predictive biomarkers not only for CEU-938 but also for additional CYP1A1-metabolized prodrugs.

From a clinical implementation perspective, FOXA1 is already a well-established biomarker, with multiple clinically validated antibodies suitable for immunohistochemistry. RAB25 is less commonly used in routine clinical practice. However, its RNA expression has been associated with favorable prognostic [43]. Accordingly, evaluating FOXA1 and RAB25 in clinical samples appears feasible using complementary approaches, including RNA-based assays and immunohistochemistry. Moreover, the overlap with the existing FOXA1-defined luminal BC stratification framework may help delineate patient subgroups that are more likely to respond to CEU-938 [44].

## 4. Materials and Methods

### 4.1. Compounds and Cell Lines

Synthesis, purification and chemical characterization of 3,4,5-trimethoxyphenyl 4-(3-butyl-2-oxoimidazolidin-1-yl)benzenesulfonate (CEU-818), 3,4,5-trimethoxyphenyl 4-(2-oxo-3-pentylimidazolidin-1-yl)benzenesulfonate (CEU-826), 3,4-dimethoxyphenyl 4-(2-oxo-3-pentylimidazolidin-1-yl)benzenesulfonate (CEU-829), 3-chlorophenyl 4-(2-oxo-3-pentylimidazolidin-1-yl)benzenesulfonate (CEU-835), 3-iodophenyl 4-(2-oxo-3-pentylimidazolidin-1-yl)benzenesulfonate (CEU-934), CEU-938, and CEU-700 have been described previously [16,30]. The specifications and sources of all chemicals used in this study are provided in Supplementary Table S2. Thirty-nine human breast cell lines were obtained at the outset of the project from the American Type Culture Collection (ATCC, Breast Cancer Cell Panel 30-4500K, Manassa, VA, USA). Cells were cultured at 37 °C in a humidified atmosphere containing 5% CO_2_ (when required), using the suppliers-recommended media (Supplementary Table S3) supplemented with 1% streptomycin and 1% penicillin (Wisent, Saint Laurent, QC, Canada). Cell lines were rapidly expanded, and all experiments were conducted using low-passage cells (<15).

### 4.2. CYP1A1 CRISPR/CAS9 and Clonal Selection

A pool of CYP1A1-knockout AU565 cells was generated using CRISPR/Cas9 technology (Synthego, Redwood City, CA, USA). Clonal selection was subsequently performed by isolating single cells and expanding individual clones, leading to the establishment of two clones: AU565^C1 *ΔCYP1A1*^ and AU565^C2 *ΔCYP1A1*^. Validation was performed by SANGER Sequencing at the CHU de Québec—Université Laval Research Center, Quebec City, QC, Canada). The inference of CRISPR Edits analysis confirmed a knockout score of 100%. CYP1A1 protein depletion was further confirmed by Western blot using CYP1A1 polyclonal antibody (Supplementary Figure S7).

### 4.3. Antiproliferative Activity

For adherent cells, antiproliferative activity was assessed using the sulforhodamine B (SRB) assay, whereas the lactate dehydrogenase (LDH) assay was employed for non-adherent cell lines. Experimental conditions were harmonized across platforms, and cross-assay benchmarking in selected cell lines confirmed comparable dose–response profiles and consistent IC_50_ values. The SRB assay was performed as described by Chavez-Alvarez et al. [30], with slight modifications. Briefly, cell seeding, treatment, staining, reading, and analysis were performed as previously described. CEU-818, CEU-826, CEU-829, CEU-835 CEU-934, CEU-938, CEU-700, Phortress (Abcam, Toronto, ON, Canada), paclitaxel (Thermo Fisher Scientific, Saint Laurent, QC, Canada), and vinblastine (Cayman Chemical, Burlington, ON, Canada) were tested at escalating concentrations up to their maximal solubility limits for either 48 or 96 h depending on the doubling time of each cell line. Moreover, cells were fixed by adding cold trichloroacetic acid (Fischer chemical, Saint Laurent, QC, Canada) directly to the wells to a final concentration of 10% (*w*/*v*), followed by incubation at 4 °C for 1.5 h. Of note, IC_50_ values were determined at both 48 h and 96 h to accommodate differences in proliferation rates across cell lines. IC_50_ values at 48 h and 96 h were highly consistent for most cell lines, indicating stable sensitivity classification. In the few instances where modest discrepancies were observed, they generally did not affect the assignment of cell lines to the responsive versus non-responsive groups.

The LDH assay was performed based on the method described by Kaja et al. [45], with minor modifications. Briefly, at the end of the treatment period, Triton X-100 (MiliporeSigma, Oakville, ON, Canada) was added to the culture medium at a final concentration of 0.9%. After shaking the plates for 10 s, the content of each well was mixed thoroughly, and 50 µL was transferred to a new plate. Media without cells were processed in parallel and served as blanks. Subsequently, 50 µL of a reaction mix (2 mM of 2-(4-iodophenyl)-3-(4-nitrophenyl)-5-phenyltetrazolium chloride (INT, TCI America, Saint Laurent, QC, Canada), 3.2 mM β-nicotinamide adenine dinucleotide sodium (MiliporeSigma), 160 mM lithium lactate (MiliporeSigma), and 15 µM 1-methoxy-5-methylphenazinium methyl sulfate (MPS, Cayman Chemical) in a 200 mM Tris buffer, pH 8.2 (BioShop, Burlington, ON, Canada) was added to each well and incubated for 30 min at room temperature. The reaction was stopped by adding 50 µL of 1% (*w*/*v*) acetic acid (BioShop) in water, and the plates were shaken for 15 s before measuring absorbance at 490 nm using a SpectraMax^®^ i3x microplate reader (Molecular Devices, San Jose, CA, USA). Optical densities were plotted against compound concentrations, and the IC_50_ and standard error of the mean (SEM) were calculated by nonlinear regression using GraphPad Prism software version 10.4.2 (GraphPad software, Boston, MA, USA).

### 4.4. Bioinformatics Analyses

Transcriptomic data were obtained from the Cancer Cell Line Encyclopedia (CCLE) project. Phase II of the CCLE expanded the initial molecular characterizations by incorporating next-generation sequencing technologies, enabling comprehensive mRNA expression profiling across 1072 cancer cell lines using RNA-seq [22]. RNA-seq data (log-transformed transcripts per million values for protein-coding genes) were downloaded from the file OmicsExpressionProteinCodingGenesTPMLogp1.csv, available via the DepMap portal (https://depmap.org/portal/ (accessed on 14 February 2024)). Quantile normalization was used to harmonize global expression distributions. Differential expression analysis was performed using the limma package in R to identify genes significantly distinguishing sensitive from non-sensitive cell lines [46]. Genes with an adjusted *p*-value < 0.05 and an absolute LFC > 1.5 were considered differentially expressed. Functional enrichment analysis was then carried out using the gproflier2 and clusterProfiler R package (version 4.5.1) [47].

### 4.5. Quantitative Real-Time PCR (RT-qPCR)

Total RNA was extracted using TRIzol reagent (Thermo Fisher Scientific) and further purified with the Monarch^®^ Spin RNA Isolation Kit (New England Biolabs, Whitby, ON, Canada), which includes an on-column DNase treatment step. RNA concentration was determined using a NanoDrop 1000 spectrophotometer (Thermo Fisher Scientific, Wilmington, DE, USA). Reverse transcription was carried out on 500 ng of RNA using M-MuLV Reverse Transcriptase (10,000 units/mL, New England Biolabs), anchored Olga (dt)_22_ primers (5 μM, Integrated DNA Technologies, Kanata, ON, Canada) and dNTP (1 μM, Bio Basic, Markham, ON, Canada). Quantitative PCR experiments were performed using the SsoAdvanced Universal SYBR Green Supermix (Bio-Rad, Saint-Laurent, QC, Canada) following the manufacturer’s two-step protocol on a CFX Connect Real-Time PCR System (Bio-Rad). Primers targeting FOXA1, RAB25, RHOV, PRKCH, and HDAC9 were used, as listed in Supplementary Table S4. Expression stability of a total of five housekeeping genes was evaluated (RPL13a, hPCBP1, TBP, B2M, ACTB), and the two most stable (RPL13a and hPCBP1) were selected for normalization. For each target gene analyzed, cDNA obtained by reverse transcription was quantified to establish standard curves using a 1:10 serial dilution. Normalization was performed by dividing the copy number of the gene of interest by the mean copy number of RPL13a and hPCBP1 in AU565 cells.

### 4.6. Western Blot

Cells were lysed in ice-cold RIPA buffer (detailed in Supplementary Table S5) supplemented with Protease Inhibitor Cocktail Set III, EDTA-Free (Fischer Scientific, Saint Laurent, QC, Canada). Protein concentration was determined using the Bio-Rad protein assay (Bio-Rad) according to the manufacturer’s instructions [48]. Samples were mixed with a 4× Laemmli buffer to a final 1× concentration and denatured at 95 °C for 5 min. Equal amounts of protein (15 µg) were separated by SDS-PAGE on a 7% or 10% acrylamide Bis-Tris gel using Stain-Free technology (2.5 min activation time) and subsequently transferred onto nitrocellulose membranes. Protein transfer was performed either by the semi-dry method using the Trans-Blot^®^ Turbo™ system (Bio-Rad) for FOXA1, RAB25, RHOV, CYP1A1 or by wet transfer in Tris-Glycine buffer containing 20% methanol (Fischer chemical) for the high-molecular-weight protein HDAC9. A complete list of the antibodies used, including their sources and dilutions, is provided in Supplementary Table S6. Total protein content was assessed using a Stain-Free imaging system (Syngene g:box chemi XR5, Cambridge, UK), and images of both gels and membranes were acquired for normalization. Membranes were incubated overnight at 4 °C with primary antibodies, followed by a 1 h incubation at room temperature with an HRP-conjugated secondary antibody. Protein bands were visualized using Immobilon^®^ ECL UltraPlus Western HRP Substrate (MilliporeSigma), and images were captured with the i system Syngene g: box chemi XR5 imaging system. Image analyses were performed using Image Lab software version 6.1 (Bio-Rad).

### 4.7. Statistics

For each experiment, at least three independent biological experiments (n ≥ 3) were performed. Statistical analyses were conducted using GraphPad Prism software version 10.4.2. Relationships in RT-qPCR and Western blot data were assessed using Spearman correlation coefficients. Comparisons between two groups were performed using unpaired two-tailed Student’s *t*-tests. A *p*-value < 0.05 was considered statistically significant for all analyses. Details of the bioinformatics analyses are provided in the corresponding sections.

## 5. Conclusions

In summary, this study identified FOXA1 and RAB25 as robust predictive biomarkers of CEU-938 activity in BC cell lines, supported by concordant evidence at both mRNA and protein levels. Collectively, our findings position CEU-938 as a compelling precision-oncology candidate, combining high target selectivity, low toxicity, and biomarker-guided patient stratification, with particular relevance to defined subsets of TNBC. Beyond CEU-938, FOXA1 and RAB25 offer a broader translational framework for the development of targeted therapies and may be applicable to other CYP1A1-activated prodrugs, including benzothiazole- and aminoflavone-based analogs. Future evaluation of FOXA1 and RAB25 expression in patient-derived breast cancer tissues will enable the identification of patients most likely to benefit from CEU-938-based therapies while also informing the broader clinical application of CYP1A1-activated prodrugs, thereby reinforcing their translational impact and precision-medicine potential.

## Figures and Tables

**Table 1:** Antiproliferative activity (IC_50_) of CEU-938, CEU-700, Phortress, paclitaxel and vinblastine in human breast cell lines at 48 h. IC_50_ values were determined using either the sulforhodamine B (SRB) assay or the lactate dehydrogenase (LDH) assay, depending on the cell line, with escalating drug concentrations up to each compound’s solubility limit. nd: not determined.

	IC_50_ (nM)				
	CEU-938	CEU-700	Phortress	Paclitaxel	Vinblastine
AU565^c1 *ΔCYP1A1*^	2000 ± 700	4 ± 1	1000 ± 600	nd	nd
AU565^c2 *ΔCYP1A1*^	4000 ± 1000	5 ± 1	500 ± 200	nd	nd
AU565	32 ± 5	10 ± 1	110 ± 15	0.8 ± 0.1	0.7 ± 0.1
184B5	>5000	80 ± 40	3 ± 2	3 ± 1	0.8 ± 0.3
BT-20	300 ± 40	2.1 ± 0.9	140 ± 30	2.1 ± 0.8	3.1 ± 0.6
BT-474	13 ± 7	1.0 ± 0.3	1400 ± 400	5 ± 2	2.5 ± 0.5
BT-549	>5000	12 ± 3	1200 ± 100	0.13 ± 0.07	1.7 ± 0.4
CAMA-1	180 ± 30	12 ± 3	600 ± 300	7 ± 2	8 ± 2
DU4475	2200 ± 900	140 ± 60	3500 ± 700	11 ± 6	60 ± 20
HCC1187	40 ± 8	2.9 ± 0.9	16 ± 7	0.5 ± 0.1	1.9 ± 0.5
HCC1395	>5000	14 ± 4	800 ± 200	1.0 ± 0.6	1.2 ± 0.4
HCC1419	1000 ± 300	40 ± 10	700 ± 200	30 ± 7	4.1 ± 0.9
HCC1500	1500 ± 300	1.5 ± 0.7	2700 ± 800	2.4 ± 0.5	0.5 ± 0.1
HCC1569	>5000	4 ± 1	2000 ± 1000	2.1 ± 0.9	1.5 ± 0.3
HCC1806	1400 ± 400	3 ± 1	400 ± 200	0.26 ± 0.08	0.4 ± 0.1
HCC1937	>5000	40 ± 9	5000 ± 100	13 ± 9	2 ± 1
HCC1954	56 ± 8	4 ± 1	440 ± 40	2.2 ± 0.8	0.4 ± 0.1
HCC202	36 ± 6	15 ± 4	60 ± 10	1.9 ± 0.3	2.0 ± 0.6
HCC38	700 ± 90	18 ± 6	54 ± 7	0.8 ± 0.1	2.9 ± 0.6
HCC70	230 ± 60	28 ± 7	80 ± 10	2.8 ± 0.6	1.5 ± 0.4
Hs 578T	>5000	19 ± 4	5000 ± 1000	0.9 ± 0.2	1.6 ± 0.2
MCF 10A	>5000	1.1 ± 0.3	1900 ± 500	0.15 ± 0.07	0.31 ± 0.07
MCF-12A	>5000	5 ± 2	3800 ± 700	0.4 ± 0.2	0.3 ± 0.1
MCF7	60 ± 10	1.2 ± 0.2	140 ± 40	1.1 ± 0.3	1.1 ± 0.2
MDA-kb2	30 ± 4	13 ± 5	1000 ± 100	3.2 ± 0.8	0.6 ± 0.1
MDA-MB-157	4000 ± 1000	0.3 ± 0.2	200 ± 100	40 ± 20	20 ± 5
MDA-MB-231	4800 ± 500	25 ± 2	6000 ± 1000	13 ± 3	2.2 ± 0.4
MDA-MB-453	90 ± 20	50 ± 10	1000 ± 90	1.5 ± 0.8	0.8 ± 0.4
MDA-MB-468	40 ± 10	3 ± 1	12 ± 9	2.0 ± 0.5	0.14 ± 0.05
SK-BR-3	26 ± 7	1.1 ± 0.6	30 ± 10	1.9 ± 0.4	0.4 ± 0.2
T47D	160 ± 50	44 ± 8	190 ± 30	2.4 ± 0.3	12 ± 5
ZR-75-1	48 ± 4	47 ± 1	6 ± 2	4 ± 2	2.90 ± 0.03
ZR-75-30	120 ± 20	3.0 ± 0.8	26 ± 5	1.9 ± 0.5	6 ± 3

**Table 2:** Antiproliferative activity (IC_50_) of CEU-938, CEU-700, Phortress, paclitaxel and vinblastine in human breast cell lines at 96 h. IC_50_ values were determined using either the sulforhodamine B (SRB) assay or the lactate dehydrogenase (LDH) assay, depending on the cell line, with escalating drug concentrations up to each compound’s solubility limit. nd: not determined.

	IC_50_ (nM)				
	CEU-938	CEU-700	Phortress	Paclitaxel	Vinblastine
AU565^c1 *ΔCYP1A1*^	2600 ± 700	8 ± 1	60 ± 10	nd	nd
AU565^c2 *ΔCYP1A1*^	1200 ± 400	2.2 ± 0.7	140 ± 40	nd	nd
AU565	32 ± 3	9 ± 1	91 ± 6	0.73 ± 0.07	0.46 ± 0.08
BT-20	200 ± 20	4.0 ± 0.7	230 ± 90	1.9 ± 0.6	2.4 ± 0.8
BT-474	32 ± 9	1.2 ± 0.2	410 ± 70	7.8 ± 1	2.6 ± 0.2
BT-483	500 ± 100	18 ± 7	270 ± 60	13 ± 4	5 ± 1
BT-549	>5000	9.0 ± 0.8	2600 ± 700	0.15 ± 0.04	1.1 ± 0.2
CAMA-1	90 ± 20	8.0 ± 0.8	230 ± 60	4.1 ± 0.9	3 ± 1
DU4475	600 ± 80	20 ± 3	7100 ± 800	12 ± 2	12 ± 2
HCC1187	36 ± 9	1.2 ± 0.2	15 ± 4	0. 41 ± 0.06	1.2 ± 0.4
HCC1395	>5000	14 ± 7	1000 ± 600	2.7 ± 0.8	0.6 ± 0.2
HCC1419	1200 ± 100	14 ± 5	220 ± 40	17 ± 3	2.9 ± 0.6
HCC1428	220 ± 50	5 ± 1	90 ± 10	1.5 ± 0.3	1.0 ± 0.2
HCC1500	1000 ± 200	1.6 ± 0.9	4500 ± 200	1.0 ± 0.2	0.42 ± 0.9
HCC1569	>5000	4 ± 1	3200 ± 300	0.8 ± 0.2	1.6 ± 0.2
HCC1806	180 ± 40	3.1 ± 0.8	150 ± 30	0.31 ± 0.04	0.43 ± 0.07
HCC1937	>5000	16 ± 4	5600 ± 600	3.9 ± 0.6	1.6 ± 0.6
HCC1954	53 ± 6	4.5 ± 0.8	290 ± 50	2.3 ± 0.5	0.36 ± 0.06
HCC2218	62 ± 9	17 ± 4	410 ± 40	5 ± 1	4 ± 1
HCC202	20 ± 6	21 ± 5	49 ± 7	1.4 ± 0.2	4.2 ± 0.7
HCC38	550 ± 40	27 ± 9	60 ± 9	1 ± 0.3	5 ± 1
HCC70	44 ± 4	47 ± 4	60 ± 16	1.2 ± 0.2	0.8 ± 0.1
Hs 578T	>5000	20 ± 4	6100 ± 500	0.78 ± 0.07	1.6 ± 0.1
MCF7	62 ± 6	1.6 ± 0.3	40 ± 8	1.6 ± 0.9	0.8 ± 0.1
MDA-kb2	17 ± 2	25 ± 5	280 ± 70	3 ± 0.5	0.6 ± 0.1
MDA-MB-134-VI	1000 ± 400	0.91 ± 0.06	2000 ± 600	3 ± 0.8	0.8 ± 0.1
MDA-MB-157	100 ± 10	45 ± 8	70 ± 40	0.8 ± 0.4	0.4 ± 0.2
MDA-MB-175-VII	90 ± 20	3.7 ± 0.9	644 ± 80	10 ± 3	3 ± 1
MDA-MB-231	4000 ± 300	33 ± 3	3000 ± 1000	3.2 ± 0.4	3 ± 1
MDA-MB-361	220 ± 50	0.7 ± 0.3	21 ± 9	1.5 ± 0.7	5.6 ± 0.1
MDA-MB-415	900 ± 200	0.9 ± 0.3	2300 ± 700	1.6 ± 0.7	1.4 ± 0.3
MDA-MB-436	1400 ± 800	8 ± 2	3900 ± 500	2.7 ± 0.6	1 ± 0.2
MDA-MB-453	100 ± 10	45 ± 8	70 ± 40	0.8 ± 0.4	0.4 ± 0.2
MDA-MB-468	20 ± 5	2.0 ± 0.8	7 ± 3	0.9 ± 0.3	0.10 ± 0.04
SK-BR-3	29 ± 0.5	3 ± 1	37 ± 9	1.6 ± 0.3	0.6 ± 0.1
T47D	73 ± 7	49 ± 6	130 ± 30	1.0 ± 0.1	3.7 ± 0.3
ZR-75-1	37 ± 3	50 ± 10	3 ± 1	1.3 ± 0.2	3 ± 1
ZR-75-30	100 ± 20	1.0 ± 0.2	19 ± 6	1.0 ± 0.3	4 ± 1

**Table 3:** IC_50_ of CEU-818, CEU-826, CEU-829, CEU-835, and CEU-934. The reference classification was based on CEU-938-responsive cell lines. IC_50_ values were determined at 48 h, except for cell lines marked with an asterisk (*), which were assessed at 96 h. Values in bold indicate a response profile that differs from that observed with CEU-938.

	CEU-818	CEU-826	CEU-829	CEU-835	CEU-934
AU565	17 ± 3	9 ± 2	29 ± 4	70 ± 10	44 ± 5
184B5	4000 ± 1000	2200 ± 700	4000 ± 1000	>5000	>5000
BT-20	700 ± 100	110 ± 20	**1100 ± 100**	**1200 ± 300**	800 ± 200
BT-474	15 ± 7	0.15 ± 0.07	2 ± 0.9	15 ± 7	10 ± 4
BT-483 *	11 ± 5	120 ± 40	**1200 ± 300**	**2300 ± 300**	**5000 ± 1000**
BT-549	>5000	>5000	>5000	>5000	>5000
CAMA-1	50 ± 20	200 ± 100	120 ± 50	200 ± 80	130 ± 60
DU4475	**200 ± 40**	>5000	>5000	>5000	>5000
HCC1187	21 ± 8	28 ± 7	400 ± 80	**1000 ± 200**	440 ± 90
HCC1395	>5000	>5000	>5000	>5000	>5000
HCC1419	>5000	>5000	>5000	>5000	3100 ± 800
HCC1428 *	700 ± 600	**>5000**	**1400 ± 700**	**2000 ± 1000**	**2100 ± 600**
HCC1500	**320 ± 90**	**800 ± 200**	1800 ± 400	2200 ± 400	2600 ± 500
HCC1569	>5000	>5000	>5000	>5000	>5000
HCC1806	**160 ± 35**	**400 ± 70**	5000 ± 1000	>5000	5000 ± 2000
HCC1937	>5000	>5000	>5000	>5000	>5000
HCC1954	10 ± 3	10 ± 2	60 ± 10	240 ± 30	130 ± 20
HCC2218 *	14 ± 4	16 ± 8	70 ± 10	180 ± 30	57 ± 9
HCC202	10 ± 2	10 ± 1	33 ± 6	90 ± 20	30 ± 9
HCC38	170 ± 40	350 ± 70	**4000 ± 1000**	**4600 ± 500**	**2500 ± 300**
HCC70	20 ± 10	100 ± 30	700 ± 200	600 ± 2000	400 ± 90
Hs 578T	>5000	>5000	>5000	>5000	>5000
MCF 10A	2300 ± 300	**900 ± 200**	**700 ± 200**	**310 ± 80**	**210 ± 70**
MCF-12A	3000 ± 1000	2200 ± 800	1900 ± 400	>5000	>5000
MCF7	40 ± 8	170 ± 60	54 ± 6	370 ± 80	110 ± 10
MDA-kb2	-	1.9 ± 0.3	35 ± 6	160 ± 20	60 ± 4
MDA-MB-134-VI *	**170 ± 20**	**120 ± 40**	**640 ± 80**	1000 ± 200	1300 ± 100
MDA-MB-157	1500 ± 300	**130 ± 50**	**300 ± 100**	**370 ± 70**	**400 ± 100**
MDA-MB-175-VII *	32 ± 5	4 ± 0.8	17 ± 4	38 ± 6	24 ± 3
MDA-MB-231	3600 ± 700	2600 ± 200	3900 ± 900	>5000	>5000
MDA-MB-361 *	16 ± 7	4.3 ± 0.8	16 ± 5	80 ± 20	53 ± 6
MDA-MB-415 *	**7 ± 2**	**0.04 ± 0.01**	**0.7 ± 0.2**	**15 ± 4**	**14 ± 3**
MDA-MB-436 *	**500 ± 200**	**200 ± 90**	**700 ± 300**	**160 ± 90**	**110 ± 70**
MDA-MB-453	-	160 ± 40	230 ± 50	**1000 ± 200**	290 ± 50
MDA-MB-468	11 ± 6	17 ± 8	500 ± 100	160 ± 60	80 ± 20
SK-BR-3	2 ± 1	2 ± 1	21 ± 7	100 ± 10	80 ± 20
T47D	12 ± 4	60 ± 20	150 ± 30	400 ± 90	180 ± 40
ZR-75-1	-	-	160 ± 30	210 ± 40	90 ± 7
ZR-75-30	11 ± 5	27 ± 8	220 ± 80	**1000 ± 200**	210 ± 40

## Data Availability

Data is contained within the article.

## Supplementary Materials

The following supporting information can be downloaded at https://www.mdpi.com/article/10.3390/ph19030357/s1, Figure S1. Phortress activity across a panel of 37 human breast cancer and non-tumorigenic cell lines (25 active cell lines). The sensitivity threshold was defined using AU565*^ΔCYP1A1^* at 48 h. Figure S2. Principal component analysis (PCA) based on CEU-938 IC50 values. PCA of global gene-expression profiles from CEU-938-responsive (red) and non-responsive (blue) cell lines. Figure S3. Representative uncropped membranes and stain-free detection of Western blots. Figure S4. Representative uncropped membranes and stain-free detection of RHOV Western blots. Figure S5. FOXA1 Western blot using sc-101058 antibody (Santa Cruz Biotechnology, Dallas, TX, USA) with stain-free detection. Figure S6. Representative uncropped membranes and stain-free detection for MCF-12A and MDA-kb2 Western blot. Figure S7. Confirmation of CYP1A1 knockout in AU565*^ΔCYP1A1^* by Western blot. Table S1. Cell lines included in the bioinformatics analyses and classification as responsive (R, pink) and non-responsive (NR, blue) to CEU-938 and Phortress, stratified by receptor subtype (HER2-positive (HER2^+^), estrogen receptor-positive (ER^+^), triple-negative breast cancer (TNBC), or non-cancerous (N)). Table S2. Specifications and sources of reagents. Table S3. Human breast cell lines and cell culture conditions. Table S4. Primers used for RT-qPCR experiments. Table S5. RIPA buffer composition used for cell lysis in Western blot experiments. Table S6. Antibodies used for protein detection in Western blots experiments. References [21,49,50,51] are cited in the Supplementary Materials.

## Abbreviations

The following abbreviations are used in this manuscript:

adj. *p*	Adjusted *p*-value
AHR	Aryl hydrocarbon receptor
BC	Breast cancer
CEU-700	3,5-Dichlorophenyl-4-(2-oxoimidazolidin-1-yl)benzenesulfonate
CEU-818	3,4,5-Trimethoxyphenyl 4-(3-butyl-2-oxoimidazolidin-1-yl)benzenesulfonate
CEU-826	3,4,5-Trimethoxyphenyl 4-(2-oxo-3-pentylimidazolidin-1-yl)benzenesulfonate
CEU-829	3,4-Dimethoxyphenyl 4-(2-oxo-3-pentylimidazolidin-1-yl)benzenesulfonate
CEU-835	3-Chlorophenyl 4-(2-oxo-3-pentylimidazolidin-1-yl)benzenesulfonate
CEU-934	3-Iodophenyl 4-(2-oxo-3-pentylimidazolidin-1-yl)benzenesulfonate
CEU-938	3,5-Dichlorophenyl 4-(2-oxo-3-pentylimidazolidin-1-yl)benzenesulfonate
CYP	Cytochrome P450
CYP1A1	Cytochrome P450 1A1
ER^+^	Estrogen receptor-positive
FOXA1	Forkhead box protein A1
HDAC9	Histone deacetylase 9
HER2^+^	Human epidermal growth factor receptor 2 of positive
LFC	Log2-fold change
PAIB-SOs	Phenyl 4-(2-oxo-3-alkylimidazolidin-1-yl)benzenesulfonates
PCA	Principal component analysis
PIB-SOs	Phenyl 4-(2-oxo-3-imidazolidin-1-yl)benzenesulfonates
PRKCH	Protein kinase C eta
RAB25	Ras-related protein 25
RHOV	Ras homolog family member V
TNBC	Triple-negative breast cancer
