# Supplementary material for: FOXA1 and RAB25 as Biomarkers of Breast Cancer Cell Response to CYP1A1-Activated Prodrugs: Insights from CEU-938
Source: Pharmaceuticals (Basel). 2026 Feb 25;19(3):357. doi: 10.3390/ph19030357 (PMC13029409; doi:10.3390/ph19030357)
Supplement: Supplementary file 1 [file pharmaceuticals-19-00357-s001.zip › pharmaceuticals-4127928-supplementary.pdf]

SUPPLEMENTARY DATA

FOXA1 and RAB25 as Biomarkers of Breast Cancer Cell Response to CYP1A1-Activated Prodrugs: Insights from CEU-938

Quentin Bruxelles [1,2,3], Geneviève Hamel-Côté [2,3], Marie-Pier Scott-Boyer [4,5], Vincent Ouellette [1,2,3], René C.-Gaudreault [2,3,5], Francine Durocher [3,4,5], Caroline Diorio [2,3,6], Arnaud Droit [3,4,5] and Sébastien Fortin [1,2,3,*]

[1] Faculté de Pharmacie, Université Laval, Québec, QC G1V 0A6, Canada; quentin.bruxelles.1@ulaval.ca (Q.B.); vincent.ouellette.2@ulaval.ca (V.O.)

[2] Axe Oncologie, Centre de Recherche du CHU de Québec—Université Laval, Québec, QC G1L 3L5, Canada; genevieve.hamel-cote@crchudequebec.ulaval.ca (G.H.C.); rene.c-gaudreault@crchudequebec.ulaval.ca (R.C.G.); caroline.diorio@crchudequebec.ulaval.ca (C.D.)

[3] Centre de Recherche sur le Cancer, Université Laval, Québec, QC G1J 0J9, Canada; francine.durocher@crchudequebec.ulaval.ca (F.D.); arnaud.Droit@crchudequebec.ulaval.ca (A.D.)

[4] Axe Endocrinologie et Néphrologie, Centre de Recherche du CHU de Québec—Université Laval, Québec, QC G1V 4G2, Canada; mariepier.scottboyer@crchudequebec.ulaval.ca (M.P.S.B.)

[5] Faculté de Médecine, Département de Médecine Moléculaire, Université Laval, Québec, QC G1V 0A6, Canada;

[6] Faculté de Médecine, Département de Médecine Sociale et Préventive, Université Laval,

Québec, QC G1V 0A6, Canada;

* Correspondence: sebastien.fortin@pha.ulaval.ca; Tel.: +1-(418)-525-4444 (ext. 52364)

Content

Figure S1. Phortress activity across a panel of 37 human breast cancer and non-tumorigenic cell lines (25 active cell lines). The sensitivity threshold was defined using AU565$^{\Delta CYP1A1}$ at 48 h. **Figure S2.** Principal component analysis (PCA) based on CEU-938 IC_{50} values. PCA of global gene-expression profiles from CEU-938-responsive (red) and non-responsive (blue) cell lines. **Figure S3.** Representative uncropped membranes and stain-free detection of Western blots. **Figure S4.** Representative uncropped membranes and stain-free detection of RHOV Western blots. **Figure S5.** FOXA1 Western blot using sc-101058 antibody (Santa Cruz Biotechnology, Dallas, TX, USA) with stain-free detection. **Figure S6.** Representative uncropped membranes and stain-free detection for MCF-12A and MDA-kb2 Western blot. **Figure S7.** Confirmation of CYP1A1 knockout in AU565$^{\Delta CYP1A1}$ by Western blot. **Table S1.** Cell lines included in the bioinformatics analyses and classification as responsive (R, pink) and non-responsive (NR, blue) to CEU-938 and Phortress, stratified by receptor subtype (HER2-positive ($HER2^+$), estrogen receptor-positive (ER^+), triple-negative breast cancer (TNBC), or non-cancerous (N)). **Table S2.** Specifications and sources of reagents. **Table S3.** Human breast cell lines and cell culture conditions. **Table S4.** Primers used for RT-qPCR experiments. **Table S5.** RIPA buffer composition used for cell lysis in Western blot experiments. **Table S6.** Antibodies used for protein detection in Western blots experiments.

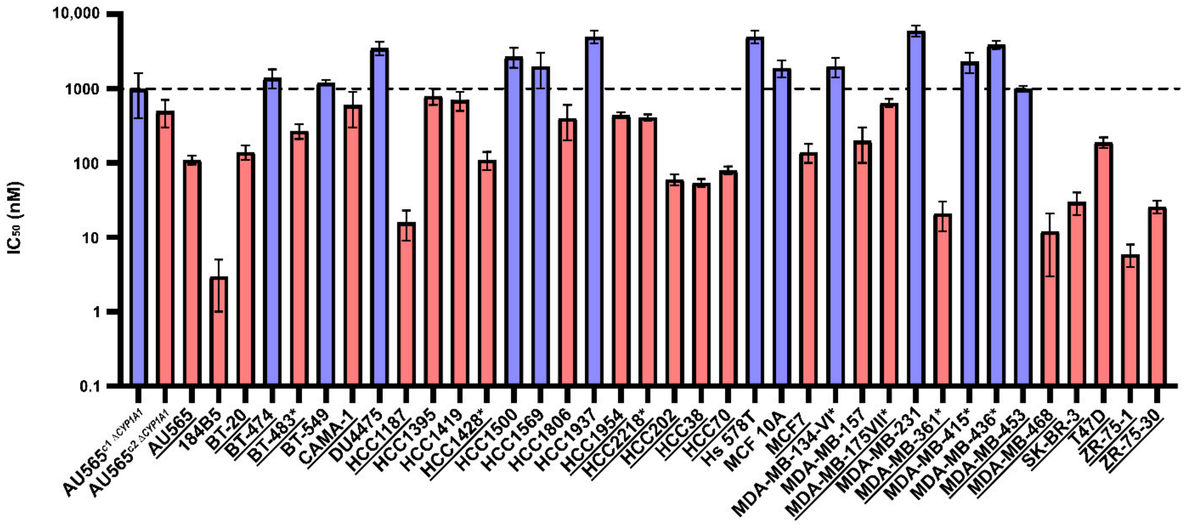

Supplementary Figure S1. Phortress activity across a panel of 37 human breast cancer and non-tumorigenic cell lines (25 active cell lines). The sensitivity threshold was defined using AU565$^{\Delta CYP1A1}$ at 48 h. IC_{50} values were assessed at 48 h, except for cell lines marked with an asterisk (*), which were evaluated at 96 h. Phortress-responsive (pink) and non-responsive (blue) cell lines for were identified. The underlying cell line models were based on CEU-938-responsive cell lines.

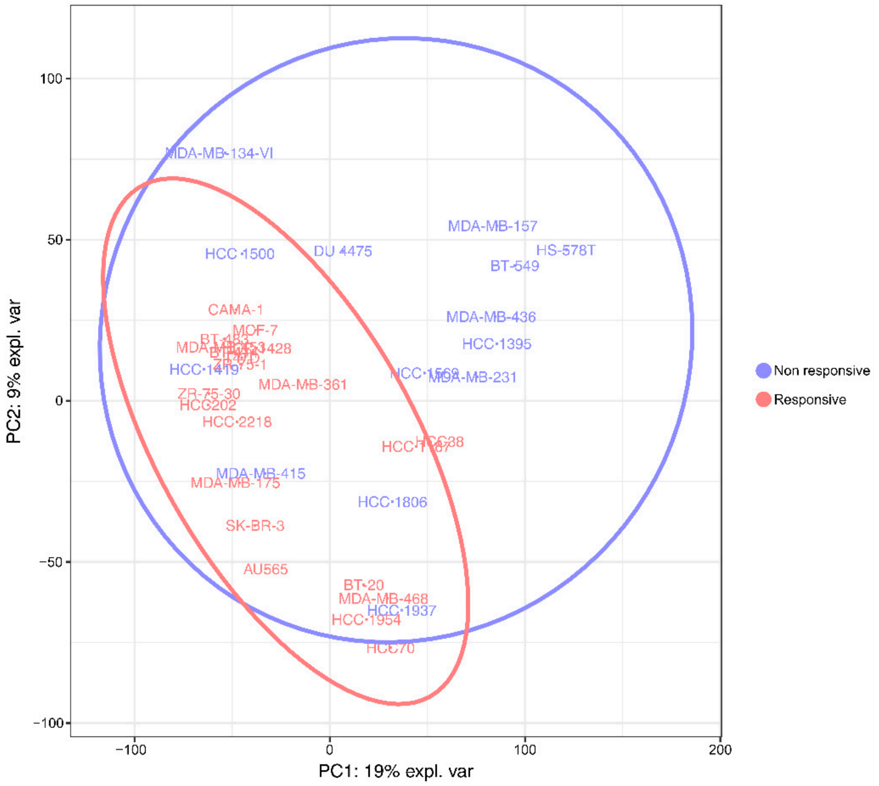

Supplementary Figure S2. Principal component analysis (PCA) based on CEU-938 IC_{50} values. PCA of global gene expression profiles from CEU-938-responsive (red) and non-responsive (blue) cell lines. The 95% confidence ellipses illustrate group clustering and reveal separation along the first two principal components, reflecting differences in global transcriptional profiles.

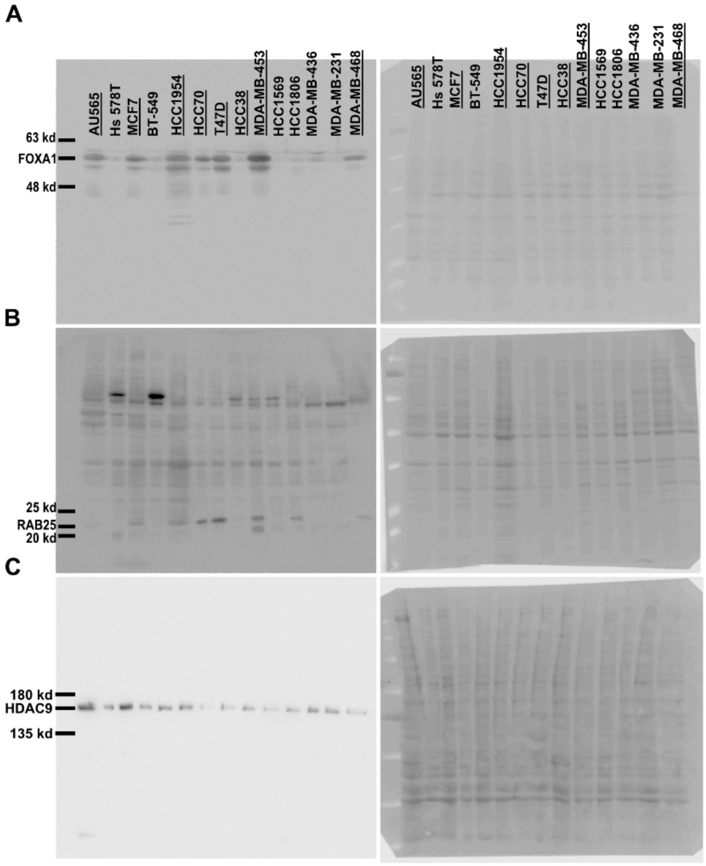

Supplementary Figure S3. Representative uncropped membranes and stain-free detection of Western blots. **A** FOXA1, **B** RAB25 and **C** HDAC9. The underlying cell line models consisted of CEU-938-responsive cell lines.

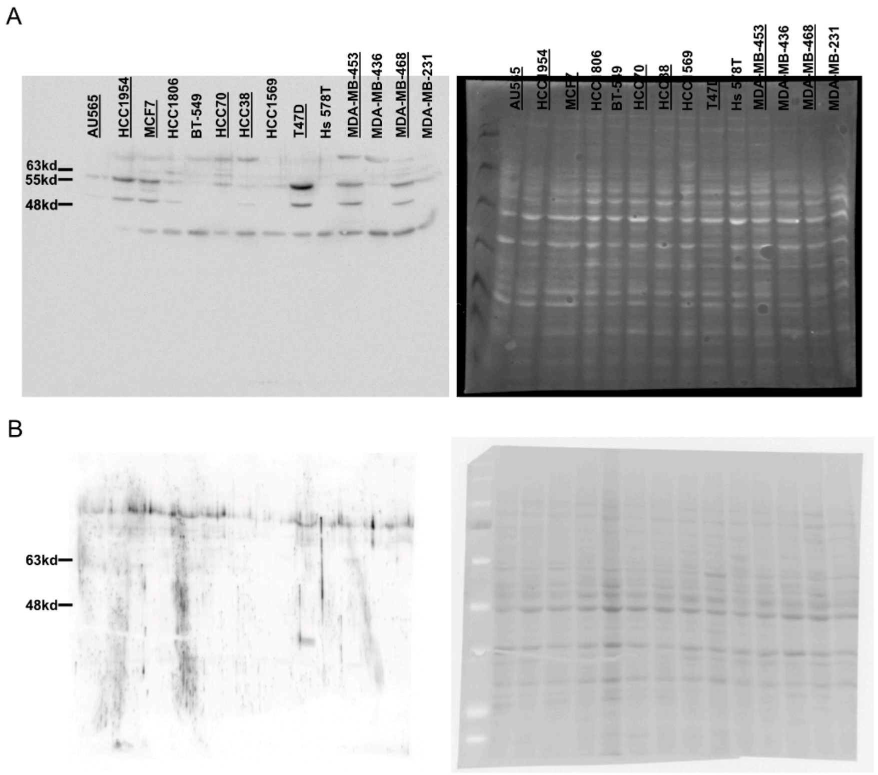

Supplementary Figure S4. Representative uncropped membranes and stain-free detection of RHOV Western blots. **A** Western blot of RHOV using the PA5-62781 antibody (Invitrogen, Burlington, ON, Canada), showing a predominant band at an incorrect molecular weight (55 kDa), together with the corresponding stain-free detection. **B** Western blot of RHOV using the OSR00321W-100UL antibody (Osenses, Keswick, Australia), with the corresponding stain-free detection. The underlying cell line models consisted of CEU-938-responsive cell lines.

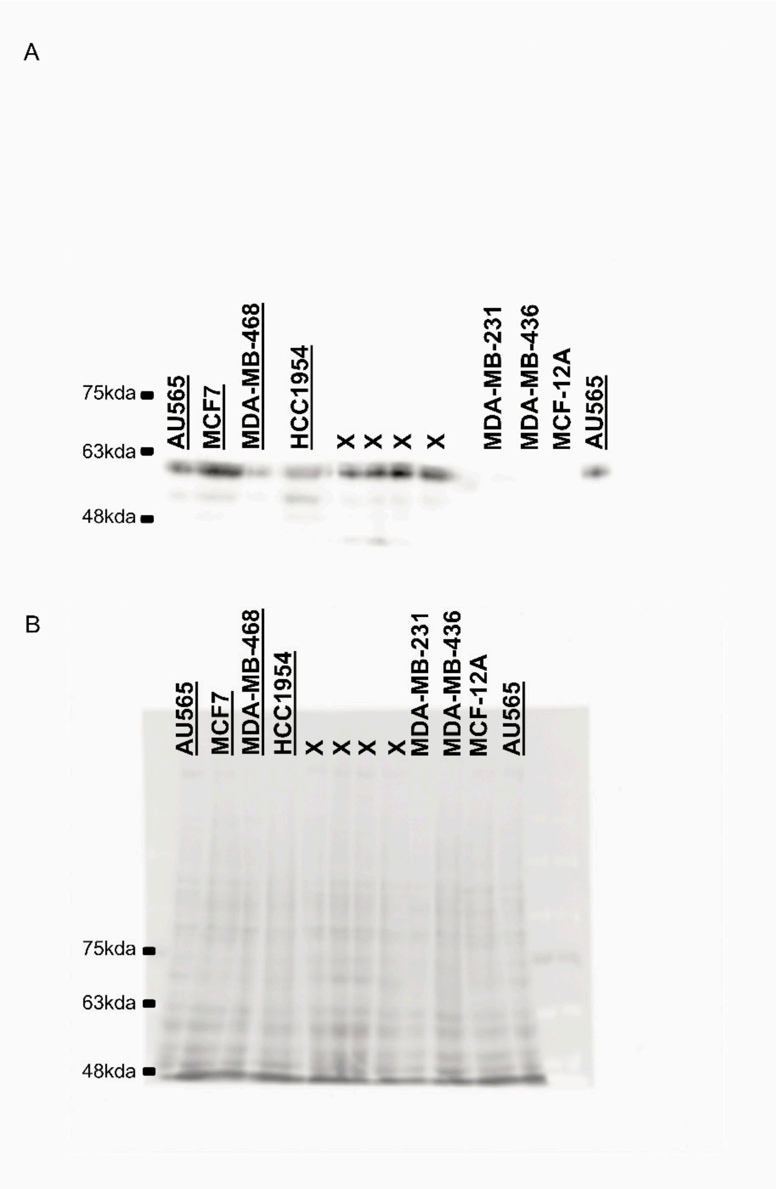

Supplementary Figure S5. FOXA1 Western blot using sc-101058 antibody (Santa Cruz Biotechnology, Dallas, TX, USA) with stain-free detection. **A** Responsive cell (AU565, MCF7, MDA-MB-468, and HCC1954), non-responsive cells (MDA-MB-231, MDA-MB-468, and MCF-12A), and positive reference control (AU565). **B** Stain-free detection. The underlying cell line models consisted of CEU-938-responsive cell lines.

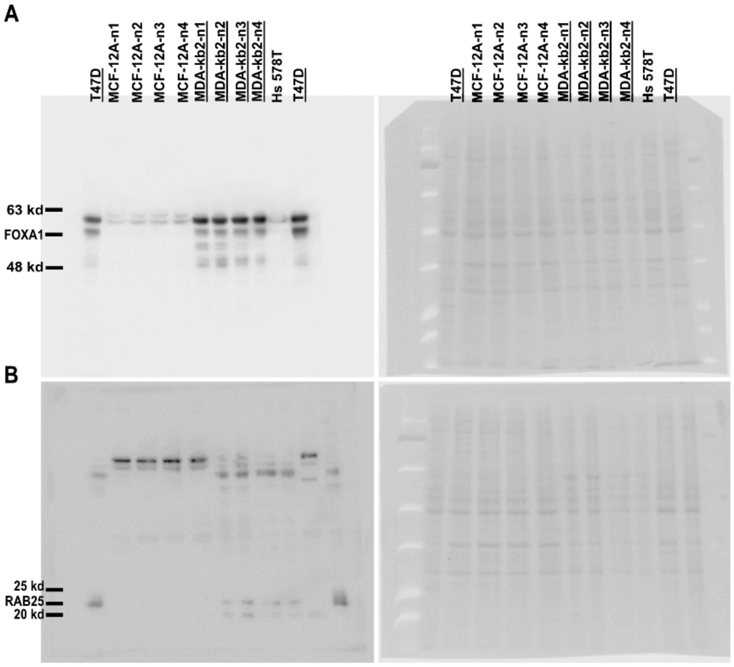

Supplementary Figure S6. Representative uncropped membranes and stain-free detection for Western blots in MCF-12A and MDA-kb2 cells. Hs 578T and T47D cell lines were used as negative and positive controls, respectively. **A** FOXA1 and **B** RAB25.

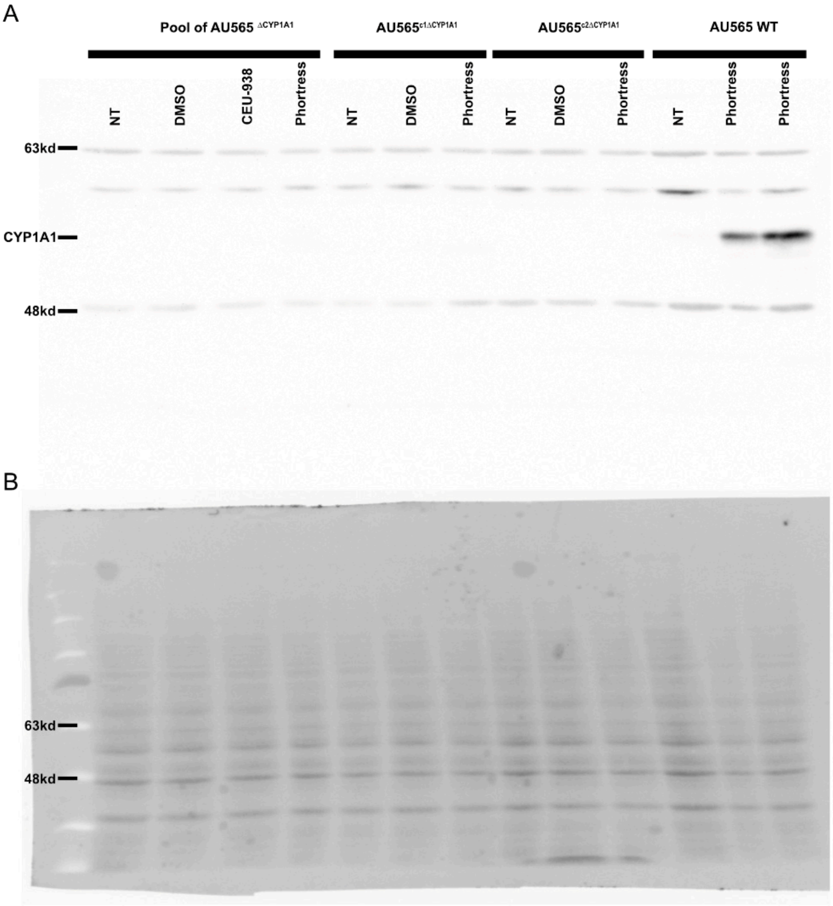

Supplementary Figure S7. Confirmation of CYP1A1 knockout in $AU565^{\Delta CYP1A1}$ by Western blot.

A Representative uncropped and **B** stain-free detection membranes.

Supplementary Table S1. Cell lines included in the bioinformatics analyses and classification as responsive (R, pink) and non-responsive (NR, blue) to CEU-938 and Phortress, stratified by receptor subtype (HER2-positive (HER2^{+}), estrogen receptor-positive (ER^{+}), triple-negative breast cancer (TNBC), or non-cancerous (N)). Classification adapted from Shari E. Smith *et al.*, 2017.

Cell lines	CEU-938	Phortress	General subtype	Bioinformatic
**BT-483***	R	R	ER^{+}	Used
**CAMA-1**	R	R	ER^{+}	Used
**HCC1428**	R	R	ER^{+}	Used
**MCF7**	R	R	ER^{+}	Used
**MDA-MB-175-VII**	R	R	ER^{+}	Used
**T47D**	R	R	ER^{+}	Used
**ZR-75-1**	R	R	ER^{+}	Used
**HCC1500**	NR	NR	ER^{+}	Used
**MDA-MB-134-VI**	NR	NR	ER^{+}	Used
**MDA-MB-415**	NR	NR	ER^{+}	Used
**AU565**	R	R	HER2^{+}	Used
**BT-474**	R	NR	HER2^{+}	Used
**HCC1954**	R	R	HER2^{+}	Used
**HCC2218**	R	R	HER2^{+}	Used
**HCC202**	R	R	HER2^{+}	Used
**MDA-MB-361**	R	R	HER2^{+}	Used
**SK-BR-3**	R	R	HER2^{+}	Used
**ZR-75-30**	R	R	HER2^{+}	Used
**HCC1419**	NR	R	HER2^{+}	Used
**HCC1569**	NR	NR	HER2^{+}	Used
**BT-20**	R	R	TNBC	Used
**HCC 1187**	R	R	TNBC	Used
**HCC38**	R	R	TNBC	Used
**HCC70**	R	R	TNBC	Used
**MDA-kb2**	R	NR	TNBC	Not used
**MDA-MB-436**	R	NR	TNBC	Used
**MDA-MB-453**	R	NR	TNBC	Used
**MDA-MB-468**	R	R	TNBC	Used
**BT-549**	NR	NR	TNBC	Used
**DU4475**	NR	NR	TNBC	Used
**HCC1395**	NR	R	TNBC	Used
**HCC1806**	NR	R	TNBC	Used

**HCC1937**	NR	NR	TNBC	Used
**Hs 578T**	NR	NR	TNBC	Used
**MDA-MB-157**	NR	R	TNBC	Used
**MDA-MB-231**	NR	NR	TNBC	Used
**184B5**	NR	R	N	Not used
**MCF 10A**	NR	NR	N	Not used
**MCF-12A**	NR	NR	N	Not used

Supplementary Table S2. Specifications and sources of reagents used.

Reagent	Supplier (distributor)	Catalog number	Location
**1-Methoxy-5-methylphenazinium methyl sulfate (MPMS)**	Cayman Chemical (Cedarlane)	21258	Burlington, ON, Canada
**2-(4-Iodophenyl)-3-(4-nitrophenyl)-5-phenyl-*2H*-tetrazolium chloride (INT)**	TCI America (Fischer Scientific)	I00671G	Saint-Laurent, QC, Canada
**Acetic acid (glacial)**	BioShop	ACE222	Burlington, ON, Canada
**Anchored oligo(dt)22 primers**	Integrated DNA Technologies (IDT)	51-01-15-09	Kanata, ON, Canada
**β-Nicotinamide adenine dinucleotide sodium (NAD)**	MiliporeSigma (Sigma-Aldrich)	N0632-1G	Oakville, ON, Canada
**β-Glycerophosphate**	Thermo Fisher Scientific	J62121.AD	Saint-Laurent, QC, Canada
**Bio-Rad protein assay kit I**	Bio-Rad	5000001	Saint-Laurent, QC, Canada
**Cholera toxin**	Sigma-Aldrich	C8052-.5MG	Oakville, ON, Canada
**Corning™ fetal bovine serum (FBS), regular**	Corning (Fisher Scientific)	MT35077CV	Saint-Laurent, QC, Canada
**dNTP mixture (10mM)**	Bio Basic	DD0056	Markham, ON, Canada
**Dulbecco's modified eagle medium (DMEM)**	Wisent	319-005 CL	Saint-Laurent, QC, Canada
**Epidermal growth factor (EGF), human**	Sigma-Aldrich	E9644	Oakville, ON, Canada
**Gibco™ horse serum, New Zealand origin**	Gibco (Thermo Fisher Scientific)	16050122	Saint-Laurent, QC, Canada
**Gibco™ human mammary epithelial cell basal medium (MEGM)**	Gibco (Fisher Scientific)	M171500	Saint-Laurent, QC, Canada
**GlutaMax™ supplement**	Gibco (Thermo Fisher Scientific)	35050061	Saint-Laurent, QC, Canada

**Ham's F12 medium**	Wisent	318-010-CL	St-Bruno , QC, Canada
**Hybri-care medium**	ATCC	46-X	Manassa, VA, USA
**Hydrocortisone**	Sigma-Aldrich	H0888-1G	Oakville, ON, Canada
**Insulin solution from bovine pancreas**	Sigma-Aldrich	I0516-5ML	Oakville, ON, Canada
**Insulin, human recombinant**	Wisent	511-016-CM	St-Bruno, QC, Canada
**Leibovitz's L-15 medium**	Wisent	323-050-CL	St-Bruno, QC, Canada
**L-Glutathione reduced**	Sigma-Aldrich	G6013-5G	Oakville, ON, Canada
**Lithium lactate**	MiliporeSigma (Sigma-Aldrich)	440469-50G	Oakville, ON, Canada
**Mammary epithelial cell growth kit**	ATCC	PCS-600-040	Manassa, VA, USA
**McCoy's 5A medium**	Wisent	317-010-CL	St-Bruno, QC, Canada
**Methanol**	Fischer chemical (Fischer Scientific)	A412-4	Saint-Laurent, QC, Canada
**MilliporeSigma™ Immobilon™ ECL UltraPlus Western HRP substrate**	MilliporeSigma (Fischer Scientific)	WBULP-100ML	Oakville, ON, Canada
**Minimum essential medium (MEM)**	Wisent	320-005-CL	Saint-Laurent, QC, Canada
**M-MuLV reverse transcriptase**	New England Biolabs	M0253S	Whitby, ON, Canada
**Monarch® spin DNA gel extraction kit**	New England Biolabs	T1120S	Whitby, ON, Canada
**Paclitaxel**	Thermo Fisher Scientific	J62734	Saint-Laurent, QC, Canada
**Penicillin-streptomycin**	Wisent	450-200 EL	Saint-Jean-Baptiste, QC, Canada
**Phortress**	Abcam	ab144638	Toronto, ON, Canada
**Protease inhibitor cocktail set III, EDTA-free**	Fischer Scientific	53-913-41ML	Saint-Laurent, QC, Canada
**RPMI 1640 medium**	Wisent	350-000-CS	Saint-Laurent, QC, Canada
**Sodium bicarbonate**	Sigma-Aldrich	S-5761	Oakville, ON, Canada
**Sodium deoxycholate**	Thermo Fisher Scientific	89904	Saint-Laurent, QC, Canada

**Sodium fluoride**	Thermo Fisher Scientific	A13019.0B	Saint-Laurent, QC, Canada
**Sodium orthovanadate**	Thermo Fisher Scientific	205330500	Saint-Laurent, QC, Canada
**Sodium pyrophosphate**	Mallinckrodt (Thermo Fisher Scientific)	6810-00N082277	Saint-Laurent, QC, Canada
**SsoAdvanced universal SYBR® green supermix**	Bio-Rad	1725274	Saint-Laurent, QC, Canada
**Trichloroacetic acid (TCA)**	Fischer chemical (Fischer Scientific)	A322-500	Saint-Laurent, QC, Canada
**Tris-base**	BioShop	TRS001	Burlington, ON, Canada
**Triton X-100**	MiliporeSigma (Sigma-Aldrich)	X100-5ML	Oakville, ON, Canada
**TRIzol reagent**	Thermo Fisher Scientific	15596026	Saint-Laurent, QC, Canada
**Vinblastine**	Cayman Chemical (Cedarlane)	11762-5	Burlington, ON, Canada

Supplementary Table S3. Human breast cell lines and cell culture conditions.

Cell line	ATCC code	Medium	Serum	Extra	Others
**184B5**	CRL-8799	MEGM	No serum	1 ng/mL cholera toxin	
**AU565**	CRL-2351	RPMI 1640	FBS 10%		
**BT-20**	HTB-19	EMEM	FBS 10%		
**BT-474**	HTB-20	Hybri-Care	FBS 10%	1.5 g/L sodium bicarbonate	
**BT-483**	HTB-121	RPMI 1640	FBS 20%	0.01 mg/mL bovine insulin	
**BT-549**	HTB-122	RPMI 1640	FBS 10%	0.023 U/mL human insulin	
**CAMA-1**	HTB-21	EMEM	FBS 10%		
**DU4475**	HTB-123	RPMI 1640	FBS 10%		
**HCC38**	CRL-2314	RPMI 1640	FBS 10%		
**HCC70**	CRL-2315	RPMI 1640	FBS 10%		
**HCC202**	CRL-2316	RPMI 1640	FBS 10%		
**HCC1187**	CRL-2322	RPMI 1640	FBS 10%		suspension and adherent
**HCC1395**	CRL-2324	RPMI 1640	FBS 10%		
**HCC1419**	CRL-2326	RPMI 1640	FBS 10%		
**HCC1428**	CRL-2327	RPMI 1640	FBS 10%		
**HCC1500**	CRL-2329	RPMI 1640	FBS 10%		
**HCC1569**	CRL-2330	RPMI 1640	FBS 10%		
**HCC1806**	CRL-2335	RPMI 1640	FBS 10%		
**HCC1937**	CRL-2336	RPMI 1640	FBS 10%		
**HCC1954**	CRL-2338	RPMI 1640	FBS 10%		
**HCC2218**	CRL-2343	RPMI 1640	FBS 10%		non-adherent
**Hs 578T**	HTB-126	DMEM	FBS 10%	0.01 mg/mL human recombinant insulin	
**MCF7**	HTB-22	EMEM	FBS 10%	0.01 mg/mL human recombinant insulin	
**MCF 10A**	CRL-10317	MEGM	No serum	100 ng/mL cholera toxin	T-75 flasks, soybean trypsin inhibitor

**MCF-12A**	CRL-10782	1:1 mixture of DMEM and Ham's F12	Horse serum 5%	20 ng/mL human EGF, 100 ng/mL cholera toxin, 0.01 mg/mL bovine insulin and 500 ng/mL hydrocortisone	
**MDA-kb2**	CRL-2713	Leibovitz's L-15	FBS 10%		No CO_2
**MDA-MB-134-VI**	HTB-23	Leibovitz's L-15	FBS 20%		No CO_2
**MDA-MB-157**	HTB-24	Leibovitz's L-15	FBS 10%		No CO_2
**MDA-MB-175-VII**	HTB-25	Leibovitz's L-15	Fetal bovine serum 10%		No CO_2
**MDA-MB-231**	HTB-26	Leibovitz's L-15	FBS 10%		No CO_2
**MDA-MB-361**	HTB-27	Leibovitz's L-15	FBS 20%		No CO_2
**MDA-MB-415**	HTB-128	Leibovitz's L-15	FBS 15%	2 mM L-glutamine, 10 µg/mL insulin, 10 µg/mL glutathione	No CO_2
**MDA-MB-436**	HTB-130	Leibovitz's L-15	FBS 15%	10 µg/mL insulin, 16 µg/mL glutathione	No CO_2
**MDA-MB-453**	HTB-131	Leibovitz's L-15	FBS 10%		No CO_2
**MDA-MB-468**	HTB-132	Leibovitz's L-15	FBS 10%		No CO_2
**SK-BR-3**	HTB-30	McCoy's 5a	FBS 10%		
**T47D**	HTB-133	RPMI 1640	FBS 10%	0.2 Units/mL bovine insulin	
**ZR-75-1**	CRL-1500	RPMI 1640	FBS 10%		
**ZR-75-30**	CRL-1504	RPMI 1640	FBS 10%		

Supplementary Table S4. Primers used for RT-qPCR experiments.

Gene	Foward (5'-3')	Reverse (5'-3')	Origin
**FOXA1**	CCAGGATGTTAGGAA CTGTGAA	CTGAGTTCATGTTGC TGACC	IDT: Hs.PT.58.1788586
**PRKCH**	CAGTTGTTCTGCTGC TTTCAG	GTGCTTCATCAAAAC GACGAG	IDT: Hs.PT.58.19109341
**RHOV**	GGACACCTTCTCTGT GCAAG	GAAGTCGGTCAAAAT CCTCCT	IDT: Hs.PT.58.40103235
**RAB25**	CTGCTGTCAAGGCTC AGAT	CATAGGTCTGGTGCT TGGTTAG	IDT: Hs.PT.58.2203953
**HDAC9**	CTGCTGTCAAGGCTC AGAT	CATAGGTCTGGTGCT TGGTTAG	IDT: Hs.PT.58.26676196
**TBP**	GCTGTTTAACTTCGC TTCCG	CAGCAAACTTCCTCA ATTCCTTG	IDT: Hs.PT.58v.39858774
**RPL13a**	CGGACCGTGCGAGGT AT	CACCATCCGCTTTTTC TTGTC	Frase S et *al*, 2022
**B2M**	ACTGAATTCACCCCC ACTGA	CCTCCATGATGCTGC TTACA	Zhang X et *al*, 2005
**ACTB**	TCAAGATCATTGCTC CTCCTGAG	ACATCTGCTGGAAGG TGGACA	Gutschner T et *al*, 2011
**hPCBP1**	ATTAATGAGATCCGC CAGATGT	CAGTGATAACCTGCC TACC	Designed by our teams

Supplementary Table S5. RIPA buffer composition used for cell lysis in Western blot experiments.

RIPA buffer	
**Reagents**	**Final concentration**
**Tris-base**	10 mM, pH 8.0
**EDTA**	1 mM
**EGTA**	0.5 mM
**Triton X-100**	1%
**Deoxycholate**	0.10%
**SDS**	0.10%
**NaCl**	140 mM
**β-Glycerophosphate**	1 mM
**Sodium pyrophosphate**	2.5 mM
**NaF**	2 mM
**Na_3V0_4**	2 mM

Supplementary Table S6. Antibodies used for protein detection in Western blots experiments.

Antibody	Reference	Dilution	Supplier (distributor)	Location
**FOXA1 Polyclonal Antibody**	PA5-27157	1/1000	Invitrogen (Thermo Fisher Scientific)	Saint Laurent, QC, Canada
**HNF-3α Antibody (Q-6, FOXA1)**	sc-101058	1/1000	Santa Cruz Biotechnolog y	Dallas, TX, USA
**Rab25 (D4P6P) XP®**	#13048	1/1000	Cell Signaling Technology (New England Biolabs)	Whitby, ON, Canada
**Anti-HDAC9 antibody [EPR5223]**	ab109446	1/1000	Abcam	Toronto, ON, Canada
**RhoV Polyclonal Antibody**	PA5-62781	1/1000	Invitrogen (Thermo Fisher Scientific)	Saint Laurent, QC, Canada
**RhoV Polyclonal Antibody**	OSR00321W-100UL	1/1000	Osenses (Thermo Fisher Scientific)	Saint Laurent, QC, Canada
**CYP1A1 Polyclonal antibody**	13241-1-AP	1/1000	Proteintech	Rosemont, IL, USA
**anti-Rabbit IgG, peroxidase-linked species-specific whole antibody (from donkey)**	45000682	1/7500	Cytiva (Fisher Scientific)	Saint Laurent, QC, Canada
